# Antioxidant and anti-inflammatory injectable hydrogel microspheres for *in situ* treatment of tendinopathy

**DOI:** 10.1093/rb/rbae007

**Published:** 2024-01-30

**Authors:** Qibin Han, Lang Bai, Yinhua Qian, Xiaoyu Zhang, Juan Wang, Jing Zhou, Wenguo Cui, Yuefeng Hao, Xing Yang

**Affiliations:** Department of Orthopedics, The Affiliated Suzhou Hospital of Nanjing Medical University, Suzhou Municipal Hospital, Gusu School, Nanjing Medical University, Suzhou 215008, P.R. China; Department of Orthopedics, The Affiliated Suzhou Hospital of Nanjing Medical University, Suzhou Municipal Hospital, Gusu School, Nanjing Medical University, Suzhou 215008, P.R. China; Department of Orthopedics, Kunshan Hospital of Traditional Chinese Medicine, Suzhou 215300, P.R. China; Department of Orthopedics, The Affiliated Suzhou Hospital of Nanjing Medical University, Suzhou Municipal Hospital, Gusu School, Nanjing Medical University, Suzhou 215008, P.R. China; Department of Orthopaedics, Shanghai Key Laboratory for Prevention and Treatment of Bone and Joint Diseases, Shanghai Institute of Traumatology and Orthopaedics, Ruijin Hospital, Shanghai Jiao Tong University School of Medicine, Shanghai 200025, P.R. China; Department of Orthopedics, The Affiliated Suzhou Hospital of Nanjing Medical University, Suzhou Municipal Hospital, Gusu School, Nanjing Medical University, Suzhou 215008, P.R. China; Department of Orthopaedics, Shanghai Key Laboratory for Prevention and Treatment of Bone and Joint Diseases, Shanghai Institute of Traumatology and Orthopaedics, Ruijin Hospital, Shanghai Jiao Tong University School of Medicine, Shanghai 200025, P.R. China; Department of Orthopedics, The Affiliated Suzhou Hospital of Nanjing Medical University, Suzhou Municipal Hospital, Gusu School, Nanjing Medical University, Suzhou 215008, P.R. China; Department of Orthopedics, The Affiliated Suzhou Hospital of Nanjing Medical University, Suzhou Municipal Hospital, Gusu School, Nanjing Medical University, Suzhou 215008, P.R. China

**Keywords:** tendinopathy, oxidative stress, inflammation, microspheres

## Abstract

Tendinopathy is a common disorder that causes local dysfunction and reduces quality of life. Recent research has indicated that alterations in the inflammatory microenvironment play a vital role in the pathogenesis of tendinopathy. Herein, injectable methacrylate gelatin (GelMA) microspheres (GM) were fabricated and loaded with heparin-dopamine conjugate (HDC) and hepatocyte growth factor (HGF). GM@HDC@HGF were designed to balance the inflammatory microenvironment by inhibiting oxidative stress and inflammation, thereby regulating extracellular matrix (ECM) metabolism and halting tendon degeneration. Combining growth factors with heparin was expected to improve the encapsulation rate and maintain the long-term efficacy of HGF. In addition, the catechol groups on dopamine have adhesion and antioxidant properties, allowing potential attachment at the injured site, and better function synergized with HGF. GM@HDC@HGF injected *in situ* in rat Achilles tendinopathy (AT) models significantly down-regulated oxidative stress and inflammation, and ameliorated ECM degradation. In conclusion, the multifunctional platform developed presents a promising alternative for the treatment of tendinopathy.

## Graphical Abstract

**Figure:**
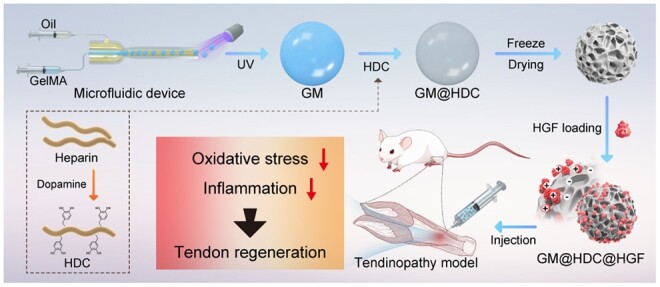

## Introduction

In tissue injuries, inflammation and oxidative stress are issues that cannot be avoided [1, 2]. Inflammatory microenvironments are crucial to the occurrence and development of various diseases. Uncontrolled inflammation can trigger a series of responses that contribute to the disorder of cellular metabolism, resulting in dysfunction and accelerated disease progression [3, 4]. Oxidative stress refers to excessive generation of reactive oxygen species (ROS) within cells and tissues that surpasses the scavenging capacity of the antioxidant system [5]. Evidence has shown that oxidative stress assumes a pathogenic function in inflammatory diseases, among which ROS are the key signaling molecules [6, 7]. Inflammation and oxidative stress are interrelated and interdependent processes. Abundant ROS are released by inflammatory cells at the inflammation site, exacerbating oxidative damage. Meanwhile, ROS and oxidative stress products also aggravate inflammation and tissue damage [2, 8]. Therefore, effective control of oxidative stress and inflammation process, promotion of tissue repair, and prevention of disease progression to the chronic phase will be the potential treatment methods.

Tendinopathy is a chronic degenerative disease characterized by the loss of normal structure and composition of the tendon collagen fibers. Sustained oxidative stress and inflammation are key factors leading to tendon degeneration [9, 10]. Following tissue injury, immune cells and activated tendon cells produce numerous pro-inflammatory cytokines such as tumor necrosis factor-alpha (TNF-α), IL-1β, and IL-6, leading to disorder of the tendon microstructure and composition [11, 12]. Injured tendons produce ROS and oxygen free radicals, which upregulate inflammation and activate tendon apoptosis and extracellular matrix (ECM) degradation [13]. The poor intrinsic healing of tendon leads to the accumulation of tissue damages and the persistence of tendinopathy [14]. To date, a variety of drugs have been explored for the anti-inflammatory and antioxidant treatment of tendinopathy such as corticosteroids, nonsteroidal anti-inflammatory drugs, platelet-rich plasma, and natural/synthetic antioxidants [15–18]. However, the effect of single drug therapy is limited and often focuses on relieving symptoms without suppressing the degeneration of affected tendons [19, 20]. In addition, as the effective dose cannot be achieved orally or by single local injection, these drugs always need to be administered repeatedly, which may cause side effects and secondary local damage [21, 22]. Therefore, it is imperative to develop a sustained-release drug delivery system with antioxidant and anti-inflammatory synergy for the treatment of tendinopathy.

Various biomaterials that have been widely researched in tendon tissue engineering possess high biocompatibility, good biomechanical properties, and modifiability [23, 24]. Since the oral administration efficiency for tendinopathy is poor owing to the characteristics of the rich matrix and lack of blood vessels in tendon tissue, researchers prefer to design materials that can work directly at the lesion site [25–27]. Many synthetic materials such as poly(lactic-co-glycolic acid) would cause chronic local inflammation during degradation, resulting in certain obstacles to tissue repair [28]. Natural materials such as gelatin and hyaluronic acid have excellent biocompatibility, but poor of mechanical strength [29]. Besides, owing to the narrow tissue gap at the tendon site, the conventional bulk materials injected *in situ* is likely to disperse because of the local activities of tendons, leading to decreased drug retention and efficacy [30]. To achieve efficient *in situ* drug release, an injectable biomaterial with properties of appropriate tissue targeting, drug protection, and high drug loading is required.

**Figure 1.**
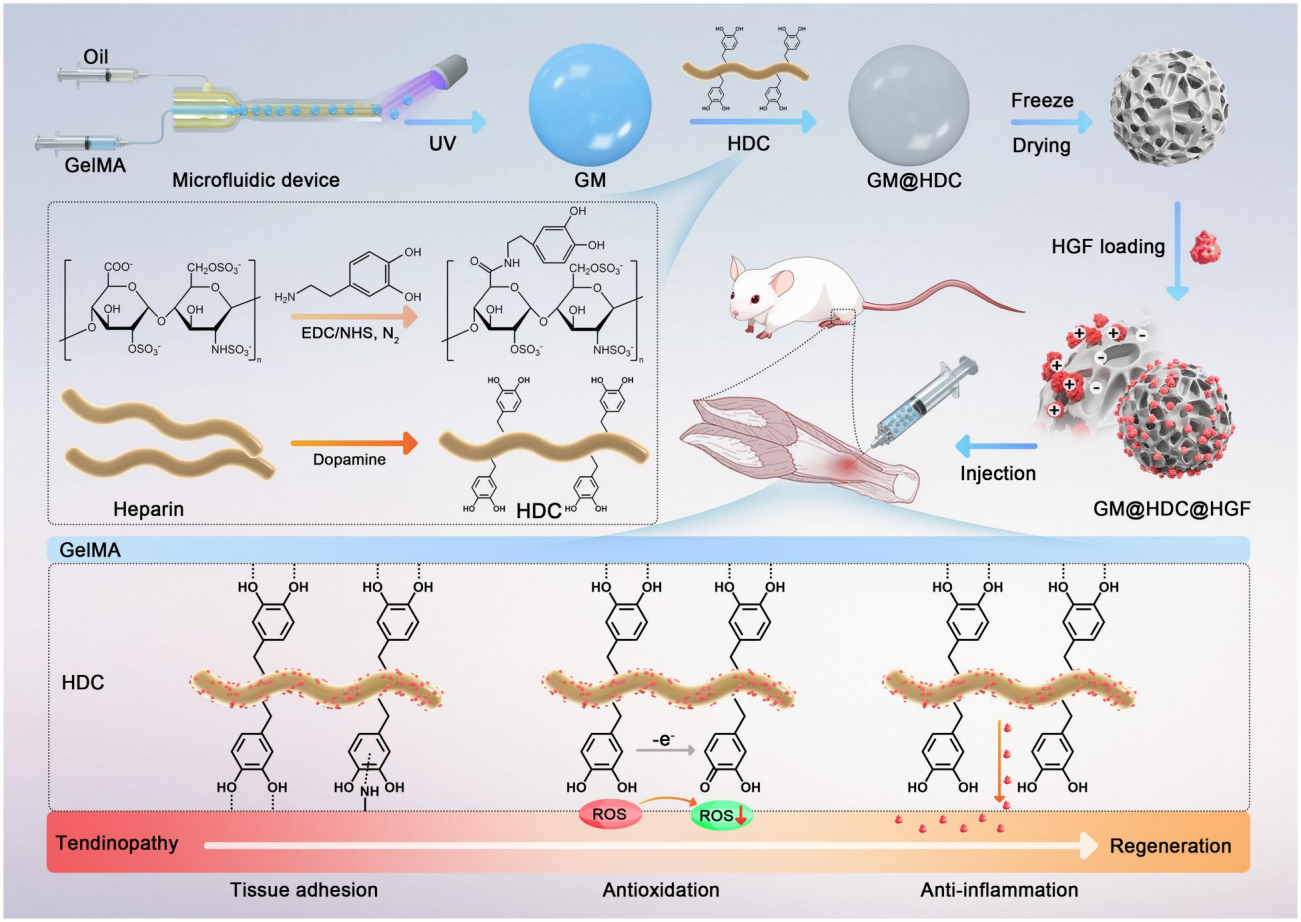
Fabrication route of GM@HDC@HGF and its properties of tissue adhesion, antioxidation and anti-inflammation for the treatment of tendinopathy.

In this study, injectable methacrylate gelatin (GelMA) microspheres (GM) functionalized with dopamine and hepatocyte growth factor (HGF) were fabricated to inhibit oxidative stress and inflammation at the pathogenic sites of tendinopathy, regulate the local tendon microenvironment, ameliorate ECM metabolic disorders, and prevent tendon degeneration. The catechol groups in dopamine have excellent adhesion and antioxidant properties and can bind firmly to the surface of GM as well as to the affected tendons after *in situ* injection, making it available to perform its physiological functions [31]. HGF is a multifunctional growth factor initially found in liver with anti-inflammatory and matrix-regulation effects [32]. Studies have shown that HGF can promote proliferation and migration of tendon-derived stem cells and mitigate inflammation in injured tendons [33, 34]. Additionally, as a heparin-binding growth factor, HGF can form stable bonds with the heparin domain to avoid inactivation by external interference [35, 36]. Taking advantage of this, dopamine was combined with heparin via an amidation reaction to form the heparin-dopamine conjugate (HDC), which was used as a bridge to efficiently bind HGF to GM. In addition, the porous properties of GM ensured a high drug loading rate. *In vitro* experiments indicated that GM@HDC@HGF significantly reduced oxidative stress and inflammation levels of tenocytes, down-regulated the expression of matrix metalloproteinases (MMPs), and promoted the balance of type I and type III collagen (COL-1, COL-3). The microspheres also showed good adhesion to tendon tissue. *In vivo* experiments in rat Achilles tendinopathy (AT) models demonstrated that the functionalized microspheres inhibited oxidative stress and inflammation, and ameliorated ECM remodeling and tendon regeneration (Figure 1).

## Materials and methods

### Materials and reagents

All chemical reagents, unless mentioned otherwise, were purchased from Aladdin. Phosphate-buffered saline (PBS), DMEM/F12, fetal bovine serum (FBS), penicillin and streptomycin were purchased from Gibco. The recombinant rat HGF protein (80429-RNAH) was purchased from Sino Biological. A cell live/dead staining kit, a cell counting kit-8 (CCK-8) and a 2,7-dichlorofluorescein diacetate (DCFH-DA) probe were purchased from Yeason. Anti-Nrf2 antibody (16396-1-AP), anti-HO-1 antibody (10701-1-AP), and anti-NQO1 antibody (67240-1-Ig) were sourced from Proteintech. Anti-COL-1 antibody (ab260043), anti-COL-3 antibody (ab7778), anti-MMP-3 antibody (ab53015), anti-MMP-13 antibody (ab39012), anti-iNOS antibody (ab178945), anti-IL-1β antibody (ab205924), anti-TNF-α antibody (ab9739), and anti-IL-6 antibody (ab233706) were sourced from Abcam. Primers for quantitative real-time polymerase chain reaction (qRT-PCR) were sourced from Generay. Enzyme-linked immunosorbent assay (ELISA) kits were sourced from Solarbio.

### Preparation of GelMA

GelMA was synthesized by the method previously reported [37]. Briefly, gelatin (20 g) was added to PBS (200 ml) and stirred at 60°C until completely dissolved. Then methacrylic anhydride (16 ml) was dripped at an average rate within 2 h through a micro syringe pump in the dark. Finally, the reaction was terminated by the addition of PBS (800 ml). The GelMA solution was dialyzed in deionized water with a dialysis bag (molecule weight: 14 kDa) for 1 week to remove unreacted substances and byproducts and kept at −20°C after lyophilization.

### Synthesis of HDC

HDC was synthesized according to the literature [38]. First, heparin sodium (500 mg) was dissolved in PBS (100 ml). Then, add N-(3-dimethylaminopropyl)-N′-ethylcarbo (EDC, 31.3 mg) and N-hydroxysuccinimide (NHS, 21.5 mg) to the solution and stir quickly for 30 min. Finally, add dopamine hydrochloride (321.3 mg) and adjust pH to 6. The reaction was maintained for 2 h, then the mixture was dialyzed in deionized water with a dialysis bag (molecule weight: 2 kDa) for 1 week to remove unreacted substances and byproducts and kept at −20°C after lyophilization.

### Synthesis of GM@HDC@HGF

GM were fabricated by a microfluidic device. About 7% (w/v) GelMA and 0.5% (w/v) photoinitiator were resolved in PBS as aqueous phase, 5% (w/w) Span80 in isopropyl sorbate was used as oil phase. Connect them to the inner and outer mouth of the coaxial nozzle (25G/21G) respectively, and adjust the internal and external injection rate to 1:30. The resulting microspheres were collected and cross-linked by ultraviolet irradiation. Deionized water and acetone were used to remove Span80 and isopropyl sorbate thrice. Then, the microspheres were immersed in HDC solution (2 mg/ml, pH = 8.5) and stirred for 12 h to obtain GM@HDC. GM@HDC@HGF were formed by loading HGF onto the microspheres via immersed adsorption.

### Characterization of HDC and microspheres

The shape and size distribution of the microspheres were captured by a bright-field microscope (BX53, Olympus). The surface morphology and microstructure of freeze-dried microspheres were observed by a scanning electron microscope (SEM, S-4800, Hitachi). The surface elements of microspheres were analyzed by energy dispersive spectroscope (EDS). The synthesis of HDC was characterized by proton nuclear magnetic resonance (^1^H NMR, Avance 400, Bruker). The charge distribution before and after the grafting of HDC and HGF was determined by Zeta potential (Zetasizer Nano ZS90, Malvern).

### Drug loading and release

GM (10 mg) and GM@HDC (10 mg) were immersed in PBS solution (1 ml) containing HGF (200 ng/ml) respectively under gentle shaking at 4°C for 12 h. Subsequently, unabsorbed HGF were removed by washing with PBS. A HGF ELISA kit was used to measure the content of HGF before and after loading. The encapsulation efficiency (%) was calculated as [(total mass of HGF−mass of residual HGF)/total mass of HGF] × 100.

After that, GM@HGF (5 mg) and GM@HDC@HGF (5 mg) were dispersed in PBS (1 ml), and then transferred to dialysis bags (molecule weight: 14 kDa), which were respectively soaked in PBS (5 ml) and dialyzed at 37°C. At predetermined time point, 1 ml supernatant was collected and stored at −80°C, and fresh PBS (1 ml) was added at the same time. The amount of released HGF was detected by ELISA.

### Biodegradability of microspheres

GM (10 mg) and GM@HDC (10 mg) were respectively immersed in PBS solution (1 ml) containing type I collagenase (2 U/ml) at 37°C. The solution was supplemented to 1 ml every other day. At predetermined time point (denoted as *T_x_*), morphological observation and residual weight measure of both GM and GM@HDC were carried out. The degradation rate (%) was calculated as [(mass at *T*_0_−mass at *T_x_*)/mass at *T*_0_]×100.

The disintegration of HDC in GM@HDC was also characterized. At predetermined time point, the supernatant was collected by centrifugation, and the optical density (OD) value of the solution at each time point was determined by a UV/visible spectrophotometer (UV-5100, METASH) at 280 nm. The shedding rate (%) of HDC was calculated as [OD value at *T_x_*/total OD value at all time points] × 100.

### Adhesion property of microspheres

Eight male Sprague Dawley rats (SD, average weight: 300–350 g) purchased from the Experimental Animal Center of Suzhou University were randomly divided into 2 groups. The experiments were approved by the Ethics Committee of the Affiliated Suzhou Hospital of Nanjing Medical University (Approval number: K-2021-067-K01). All animal procedures were performed in accordance with the National Institutes of Health Guide for Care and Use of Laboratory Animals. After anesthesia, GM or GM@HDC (50 μl, 10 mg/ml) were injected into their right Achilles tendons. The rats were sacrificed after 24 h of free movement. The right ankle joints of the rats were carefully exposed to observe the adhesion of microspheres to Achilles tendons. Further, the left Achilles tendons were separated and mixed with excessive GM or GM@HDC at 37°C for 30 min. Then the isolated Achilles tendons were carefully taken out and observed under a stereomicroscope (Stemi 508, Zeiss).

### Tenocytes isolation and culture

The primary tenocytes were isolated from Achilles tendons of SD rats aged 4–6 weeks. Achilles tendons were cut to 1 mm tissues under sterile conditions and digest in PBS solution containing collagenase (2 U/ml) at 37°C for 4 h. The solution was then removed and the tendon tissues were transferred to a cell culture dish and cultured in a 37°C incubator with 5% CO_2_ using DMEM/F12 medium containing 10% serum and 100 U/ml penicillin and streptomycin. Three to five days later, the primary tendon cells could be seen spreading out of the tendon tissues. The medium was changed every other day. In this study, tenocytes with low passage times of P_2_–P_4_ were used.

### Biocompatibility tests

To investigate the cytotoxicity of microspheres, tenocytes (5 × 10^3^/well) were incubated on 24-well plates and co-cultured with microspheres. On 1, 3, and 5 days, tenocytes were stained with a live/dead staining kit for 20 min. Then the tenocytes were photographed under a fluorescence microscope (DM13000B, Leica). The living cells were stained green and the dead cells were red. The proliferative activity of tenocytes was further evaluated using CCK-8. In brief, tenocytes (2 × 10^3^/well) were incubated on 96-well plates and co-cultured with microspheres. On 1, 3, and 5 days, CCK-8 reagent (10 μl) was added to each well and subsequently incubated at 37°C for 2 h. The absorbance was measured using a microplate reader (Varioskan LUX, Thermo) at 450 nm.

### Antioxidant activity test

As reported previously [39], the 2,2-diphenyl-1-picrylhydrazyl (DPPH) radical was used to test the antioxidant activities of GM, GM@HDC, and GM@HDC@HGF. First, DPPH (4 mg) was dissolved in methanol solution (100 ml). Then the microspheres (1 mg) were added to the solution (4 ml) for an antioxidant reaction at 37°C in the dark. At predetermined time point, the reaction solution was extracted and the absorbance was quantified at 516 nm. The DPPH scavenging efficiency (%) was calculated as [(OD value at *T*_0_−OD value at *T_x_*)/OD value at *T*_0_]×100.

### Cellular ROS scavenging activity

The ROS levels in tenocytes were detected through the utilization of a DCFH-DA probe. Tenocytes (2 × 10^4^/well) were incubated on 24-well plates and co-cultured with GM, GM@HDC, and GM@HDC@HGF. After 12 h, H_2_O_2_ (100 μM) were added to the medium. After another 4 h, DCFH-DA (10 μM) was added and incubated at 37°C for 20 min in the dark. The ROS level of each group was detected by fluorescence microscopy and flow cytometry.

### Western-blot assay

Total protein was extracted using lysates containing protease inhibitors and phosphatase inhibitors. Protein samples were isolated via SDS-PAGE, and the target proteins were subsequently transferred onto 0.45 μm PVDF membranes. These membranes were incubated with 5% skim milk at room temperature for 1 h and primary antibodies at 4°C overnight. After being washed thrice, the membranes were incubated with specific secondary antibodies for 1 h. Protein signal was detected by a chemiluminescence system (5200 Multi, Tanon).

### Quantitative real-time PCR assay

Tenocytes (1 × 10^5^/well) were incubated on 6-well plates, treated with lipopolysaccharide (LPS, 1 μg/ml) for 24 h. Then GM, GM@HDC, and GM@HDC@HGF were added in groups and co-cultured for 72 h. Total RNA was extracted using Trizol reagent. The mRNA expression levels of COL-1, COL-3, MMP-3, MMP-13, iNOS, TNF-α, IL-1β and IL-6 in different groups were detected by qRT-PCR using Lightcycler 480II (Roche) and normalized with GAPDH. The primer sequences of the genes are listed in Table 1 below.

**Table 1.** Primer sequences

Gene	Primer	Sequence (5′–3′)
COL-1	Forward	TGGAAACAGACCAACAACCCA
	Reverse	ATTTGAAGGTGCTGGGTAGGG
COL-3	Forward	CGGGCAAGAATGGAGCAAAG
	Reverse	ACCAGGGAAACCCATGACAC
MMP-3	Forward	ATGCAGGGAAAGTGACCCAC
	Reverse	CGACGCCCTCCATGAAAAGA
MMP-13	Forward	ACCATCCTGTGACTCTTGCG
	Reverse	TTCACCCACATCAGGCACTC
iNOS	Forward	TGGGTGAAAGCGGTGTTCTT
	Reverse	TAGCGCTTCCGACTTCCTTG
IL-1β	Forward	GAGTCTGCACAGTTCCCCAA
	Reverse	ATGTCCCGACCATTGCTGTT
TNF-α	Forward	CGTCAGCCGATTTGCCATTT
	Reverse	CCCAGAGCCACAATTCCCTT
IL-6	Forward	AGCCACTGCCTTCCCTACTT
	Reverse	ACTCCAGAAGACCAGAGCAGA
GAPDH	Forward	ACTCTACCCACGGCAAGTTC
	Reverse	TGGGTTTCCCGTTGATGACC

### Immunofluorescence staining assay

Tenocytes (5 × 10^3^/well) were incubated on the cover glass of 24-well plate and treated with LPS (1 μg/ml) for 24 h. Then GM, GM@HDC, and GM@HDC@HGF were added in groups and co-cultured for 72 h. The tenocytes were fixed with 4% paraformaldehyde for 15 min, then permeabilized with 0.1% Triton X-100 for 10 min and incubated with the blocking solution for 1 h, following by incubation with the primary antibodies at 4°C overnight. After being washed thrice, the tenocytes were incubated with alexafluor-conjugated secondary antibodies for 1 h. Cytoskeleton and nucleus were stained with phalloidin and DAPI for 20 min, respectively. At last, the images were observed and photographed using a laser confocal scanning microscope (LSM900, Zeiss).

### *In vivo* animal experiments

A total of 60 male SD rats (average weight: 350–400 g) were used for *in vivo* experiments. After anesthesia, the rats were injected with type I collagenase solution (100 μl, 5 mg/ml) 5 mm above the right heel. After 7 days, the rat AT models were successfully established. The rats were randomly divided into 5 groups: control group, tendinopathy group, GM group, GM@HDC group and GM@HDC@HGF group. Except the control group, all other groups received injection.

The rats of the control group and tendinopathy group received no treatment, while the other 3 groups received equal injection of GM, GM@HDC and GM@HDC@HGF (100 μl, 10 mg/ml), respectively. After 1 week of treatment, half of the rats of each group were sacrificed, the right Achilles tendons were isolated and prepared for H&E, Masson, and ROS frozen staining. At the same time, the blood of the rats was extracted and serum was separated for ELISA detection of IL-1β, TNF-α and IL-6. After 4 weeks of treatment, the remaining half of the rats were sacrificed and the Achilles tendons were isolated for H&E, Masson, COL-1 and COL-3 immunohistochemical staining. Meanwhile, protein of each group was extracted for Western blot of COL-1, COL-3, MMP-3, and MMP-13.

### Statistical analysis

GraphPadPrism8.0 and ImageJ were used for data analysis and graph processing. The experimental data were analyzed by two-sided *t*-test or one-way ANOVA and expressed as mean ± SD. *P *<* *0.05 was considered to be statistically different.

## Results and discussion

### Preparation and characterization of GM@HDC@HGF

**Figure 2.**
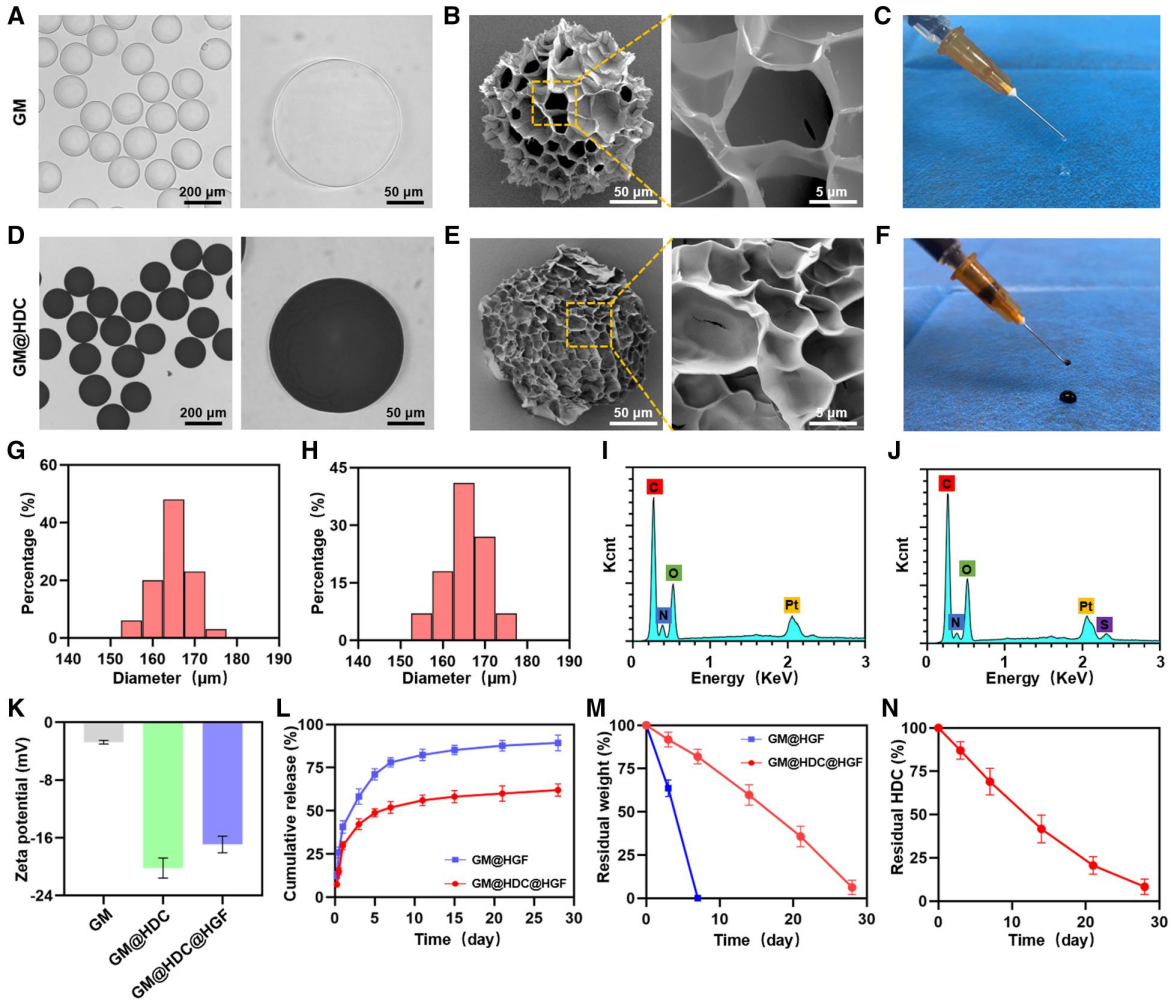
Characterization of functionalized microspheres. (**A**) Bright field image of GM. (**B**) SEM image of GM. (**C**) Demonstration of the injectability of GM. (**D**) Bright field image of GM@HDC. (**E**) SEM image of GM@HDC. (**F**) Demonstration of the injectability of GM@HDC. (**G, H**) Particle size distribution of GM and GM@HDC. (**I, J**) EDS spectrum of GM and GM@HDC. (**K**) Zeta potential of microspheres before and after grafting HDC and HGF (*n* = 3). (**L**) release curves of HGF from GM and GM@HDC (*n* = 3). (**M**) Residual mass of microspheres during degradation (*n* = 3). (**N**) Residual HDC of GM@HDC during degradation (*n* = 3).

GelMA hydrogel was chosen to form the main body of the injectable microspheres, which is characterized by excellent biocompatibility, low immunogenicity, and low cost and has therefore attracted significant attention in the field of biomedical tissue engineering materials [40–42]. We synthesized GelMA according to previously reported methods, and successfully prepared GM via microfluidic technology. GM appeared white under a light microscope and had an average diameter of 164.85 ± 4.46 µm (Figure 2A and G). After lyophilization, the GM particle size decreased and a loose porous structure was observed by SEM (Figure 2B), allowing GM a large specific surface area and increased drug loading efficiency. GM could be smoothly injected through a 29G diameter needle without obstruction, which demonstrated that it had good injectability and could be used for minimally invasive injection therapy (Figure 2C).

Heparin and dopamine were amidated to synthesize HDC. The ^1^H NMR results indicated successful grafting. The peak around 6–7 ppm is assigned to the benzene ring in dopamine (Supplementary Figure S1). When GM and HDC were stirred in alkaline solution (pH = 8.5), HDC polymerized on the GM surface to form gray black microspheres GM@HDC with an average diameter of 165.45 ± 5.03 µm (Figure 2D and H). SEM and injection tests showed that the surface modification with HDC did not alter the porous structure or injectability of the microspheres (Figure 2E and F). EDS was carried out to verify the HDC graft (Figure 2I and J). Compared with GM, the results for GM@HDC showed a new sulfur element peak specific to heparin, which confirmed the successful grafting of HDC to the surface of GM.

HGF was physically blended with the microspheres. By determining the HGF content in the solution before and after mixing, it was concluded that the loading capacity of HGF on GM was approximately 142.68 ± 3.73 ng/10 mg (encapsulation rate of HGF approximately 71%), while that on GM@HDC was 192.34 ± 2.41 ng/10 mg (encapsulation rate of HGF approximately 96%). Zeta potential characterization was conducted to verify the successful adsorption of HGF (Figure 2K). The absolute zeta potential of GM@HDC was significantly higher than that of GM owing to the strong negative charge of heparin. The absolute value of GM@HDC@HGF potential decreased slightly due to the absorption of HGF, which neutralized some of the charge. These results were consistent with a previous study showing that HGF has a strong affinity for heparin molecules [35].

The sustained release of HGF from the microspheres was investigated. Figure 2L shows the release curve of HGF from GM@HGF and GM@HDC@HGF. On the first day, sudden release of HGF was observed for both systems. The HGF release amount for GM@HGF and GM@HDC@HGF was 40.73 ± 2.42% and 29.90 ± 1.93%, respectively. The release efficiency then showed a slow decrease, and the HGF release reached a plateau approximately 14 days later. Within 28 days, the HGF release of the two groups was 89.34 ± 3.43% and 62.02 ± 1.79%, respectively. The results indicated that GM@HDC had better long-term sustained release of HGF than GM.

### Degradation and adhesion of functionalized microspheres

The retention efficiency of microspheres at tendon tissue is an important factor for *in situ* administration. Owing to the long course of tendinopathy, the biodegradation of the ideal vector should be relatively slow [9]. Although GelMA has good biocompatibility, it can be easily affected by various enzymes *in vivo* and rapidly degrades [43]. Figure 2M shows the degradation curve of GM before and after HDC grafting. GM were completely degraded within 7 days (Supplementary Figure S2A), which was not consistent with the pathological process of tendinopathy. Notably, GM@HDC showed constant and slow degradation over 28 days (Supplementary Figure S2B). The polymeric coating of HDC on the GM surface was designed to enhance the physicochemical properties of the microspheres and delay the exposure of the internal structure, thus prolonging the degradation of the microspheres [44]. As the microspheres slowly degraded, the HDC grafted on their surface gradually broke down. The shedding rates of HDC in different time periods were measured by spectrophotometry (Figure 2N). During the former 14 days of degradation, 58.31 ± 6.54% of the HDC was shed from the GM@HDC surface. After 28 days, HDC was almost completely shed with the degradation of the microspheres.

Tendons are tissues that connect bones to muscles and play an important role in joint movement [45]. Frequent partial activity can make injected microspheres be displaced from the injured area, thus reducing the delivery efficacy. The addition of the catechol groups gives GM@HDC@HGF excellent adhesion properties, allowing it to adhere firmly to affected tendons and increasing the *in situ* retention efficiency of the microspheres [28, 46]. The adhesion properties of the microspheres were tested. First, equal amounts of GM and GM@HDC were locally injected into the Achilles tendons of the rats. After 24 h, the Achilles tendons were carefully exposed and the local distribution of microspheres on the tendon tissues was observed (Supplementary Figure S3A). It can be seen that GM@HDC were closely clustered at the injection site and enriched around the Achilles tendon. GM partially diffused into the soft tissue space on both sides of the Achilles tendon. The dispersal of GM was caused by partial activity of the Achilles tendon due to the movement of the ankle joint, while GM@HDC were clustered *in situ* owing to its adhesion property. Furthermore, fresh isolated rat Achilles tendons were immersed with excessive GM and GM@HDC. After 30 min, the Achilles tendons were taken out and observed (Supplementary Figure S3B). Only a few GM were attached to the surface of the isolated Achilles tendon, compared with a large amount of GM@HDC that covered almost the entire tissue surface. These results indicated that HDC functionalized microspheres exhibited prolonged degradation and excellent adhesion, and were suitable for *in situ* injection therapy for tendinopathy.

### *In vitro* biocompatibility and cell proliferation

**Figure 3.**
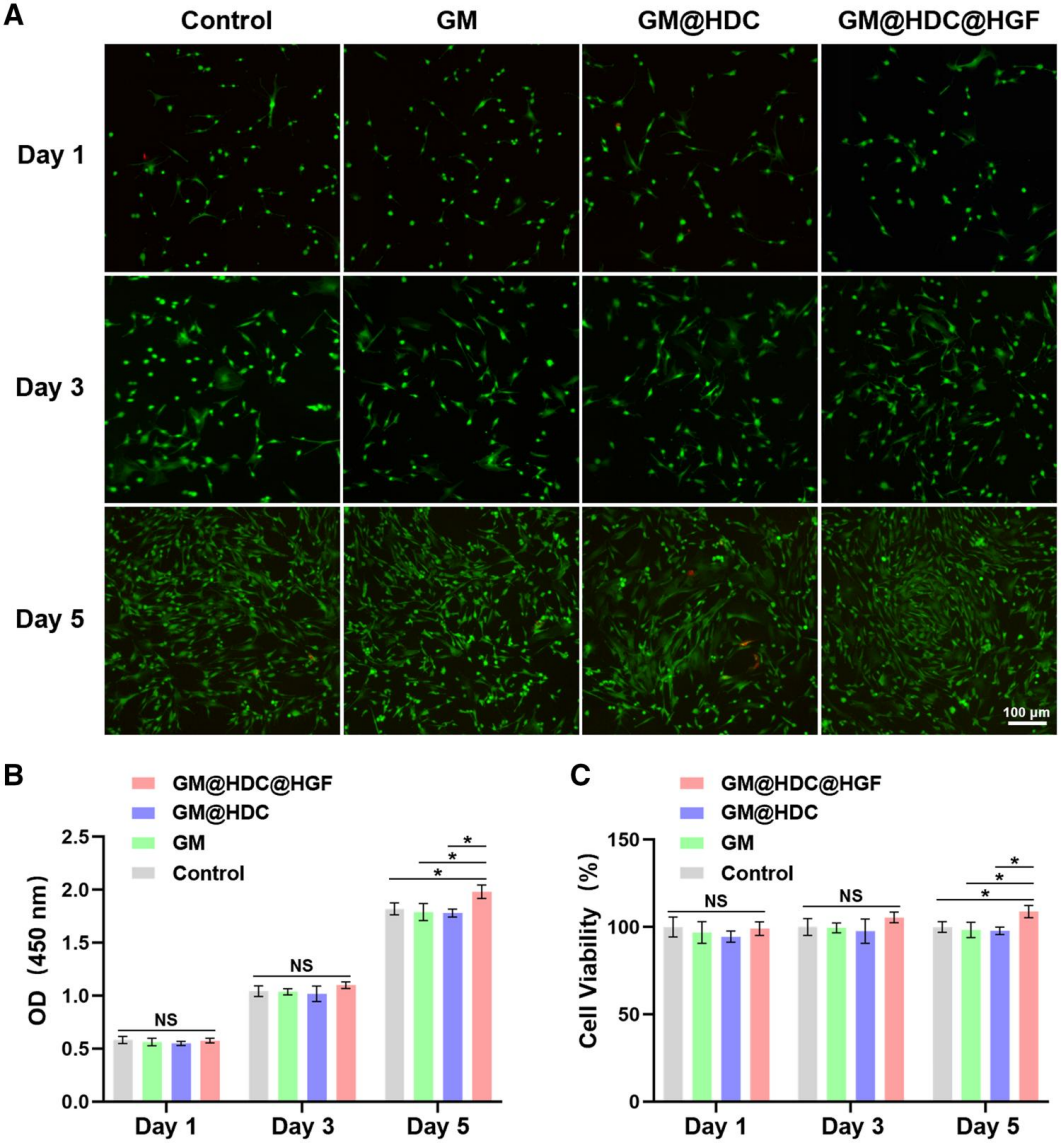
*In vitro* biocompatibility of microspheres. (**A**) Representative fluorescence live/dead staining images of tenocytes co-cultured with GM, GM@HDC, and GM@HDC@HGF. (**B, C**) The OD value at 450 nm and cell viability of tenocytes co-cultured with GM, GM@HDC, and GM@HDC@HGF (*n* = 3, NS, no significance, *P <* *0.05).

The *in vitro* biocompatibility of GM, GM@HDC, and GM@HDC@HGF was investigated to evaluate the prospective clinical utilization of the functionalized microspheres. The biocompatibility of each group was evaluated by live/dead staining and CCK-8 after co-cultured with tenocytes for 1, 3, and 5 days. As shown in Figure 3A, there were few dead cells in the field after live/dead staining over the culture period. CCK-8 quantitative analysis showed that there was no significant difference in cell proliferation activity among all groups at day 1 and 3. At day 5, the cell proliferation activity of the GM@HDC@HGF group was found to be slightly better than those of the other groups (Figure 3B and C). This may be a result of the cell proliferation effect of released HGF. The results indicated that the functionalized microspheres had excellent biocompatibility, and GM@HDC@HGF could promote the proliferation of tenocytes.

### *In vitro* antioxidant properties

In the AT microenvironment, inflammatory reaction and post-injury stress can lead to high ROS production [7]. An early high ROS level is a key factor in AT aggravation and persistence. The catechol group has been shown to be an efficient scavenger of ROS [47]. The antioxidant activity of the functionalized microspheres was evaluated using the DPPH radical scavenging test. The same amount of GM, GM@HDC, or GM@HDC@HGF were mixed with DPPH solution and allowed to react in the dark for 20, 40, 60, 80, 100, and 120 min, then the DPPH scavenging efficiency was calculated. As shown in Supplementary Figure S4, the DPPH scavenging efficiency of the 3 groups increased with time. The DPPH scavenging efficiency of the GM group was only 35.67 ± 2.52% at 120 min. However, the efficiency of GM@HDC (76.22 ± 3.13%) and GM@HDC@HGF (79.86 ± 3.08%) were both close to 80% at 120 min, and shared similar trends. This indicated that HDC functionalized microspheres had good antioxidant properties.

**Figure 4.**
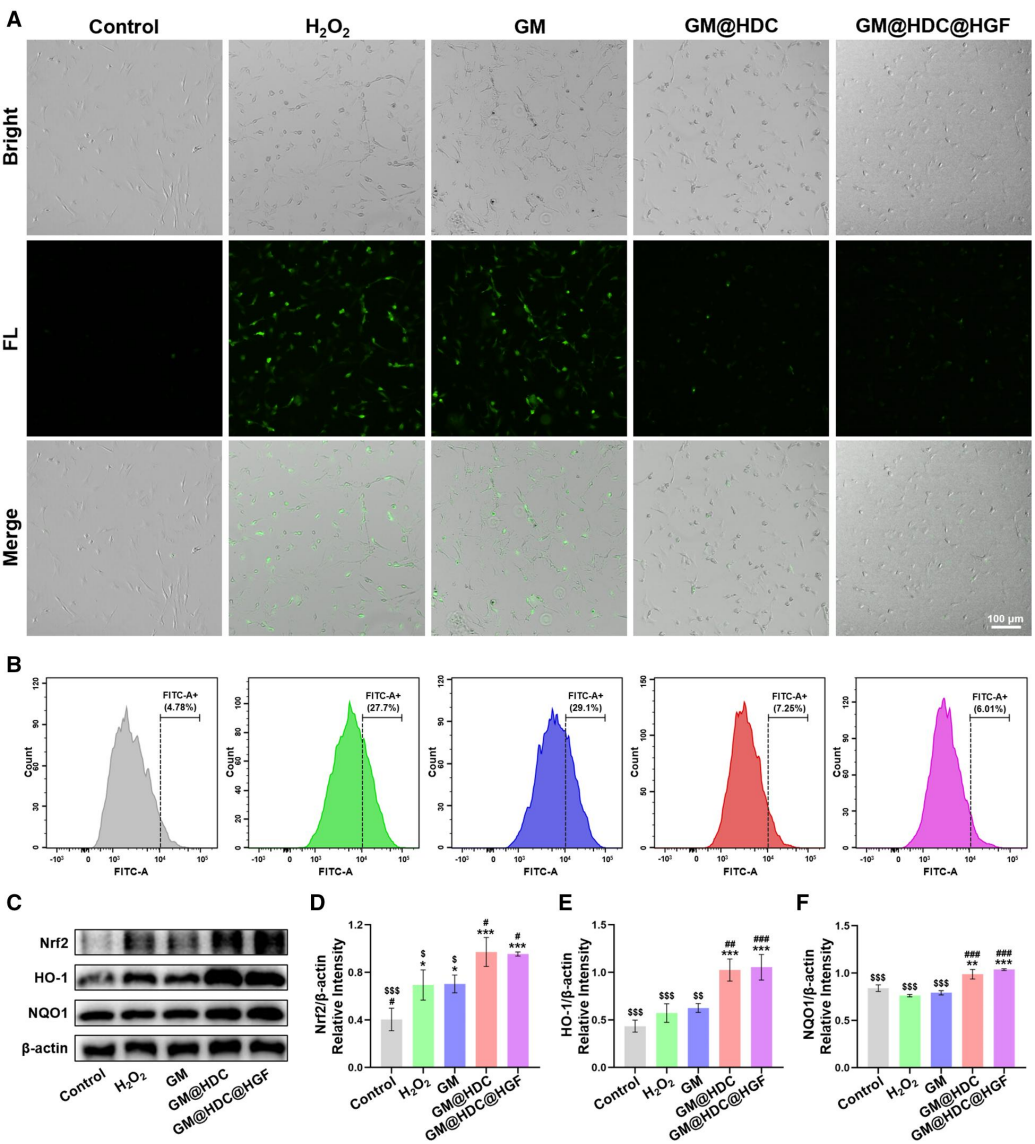
*In vitro* antioxidant property of microspheres. (**A**) Intracellular ROS-scavenging performance of tenocytes co-cultured with GM, GM@HDC, and GM@HDC@HGF. (**B**) Flow cytometric analysis of (DCFH-DA)-labeled cells in the FITC-A channel of GM, GM@HDC, and GM@HDC@HGF. Bars on the right span the stained ROS-presenting subpopulations and the percentage is shown. (**C**) Western blot of antioxidant proteins. (**D–F**) Semi-quantitative analysis of expression of antioxidant proteins (*n* = 3, */**/***, #/##/### and $/$$/$$$ indicated *P *<* *0.05/*P *<* *0.01/*P *<* *0.001 compared to the control, H_2_O_2_ and GM@HDC@HGF groups, respectively).

Microspheres of the different groups were co-cultured with H_2_O_2_-induced tenocytes to investigate their ROS scavenging properties *in vitro*. A DCFH-DA probe was used to determine the intracellular ROS level in each group (Figure 4A). Fluorescence microscopy showed that the intracellular ROS level in the H_2_O_2_ group increased significantly. The intracellular ROS level was similar to the H_2_O_2_ group after co-cultured with GM while decreased significantly after co-cultured with GM@HDC and GM@HDC@HGF. Flow cytometry results showed that in the GM@HDC and GM@HDC@HGF groups, the positive rates of intracellular ROS were 6.88 ± 0.63% and 5.93 ± 0.05%, compared with 26.77 ± 3.37% in the H_2_O_2_ group (Figure 4B). In addition, cell proteins of each group were extracted. The results of Western blot showed that the expression of oxidative stress regulatory molecule Nrf2 and its downstream proteins, HO-1 and NQO1, were significantly increased in the GM@HDC and GM@HDC@HGF groups (Figure 4C). These findings indicated that HDC functionalized microspheres effectively prevented cell damage caused by excessive ROS under oxidative stress.

### *In vitro* anti-inflammation properties

**Figure 5.**
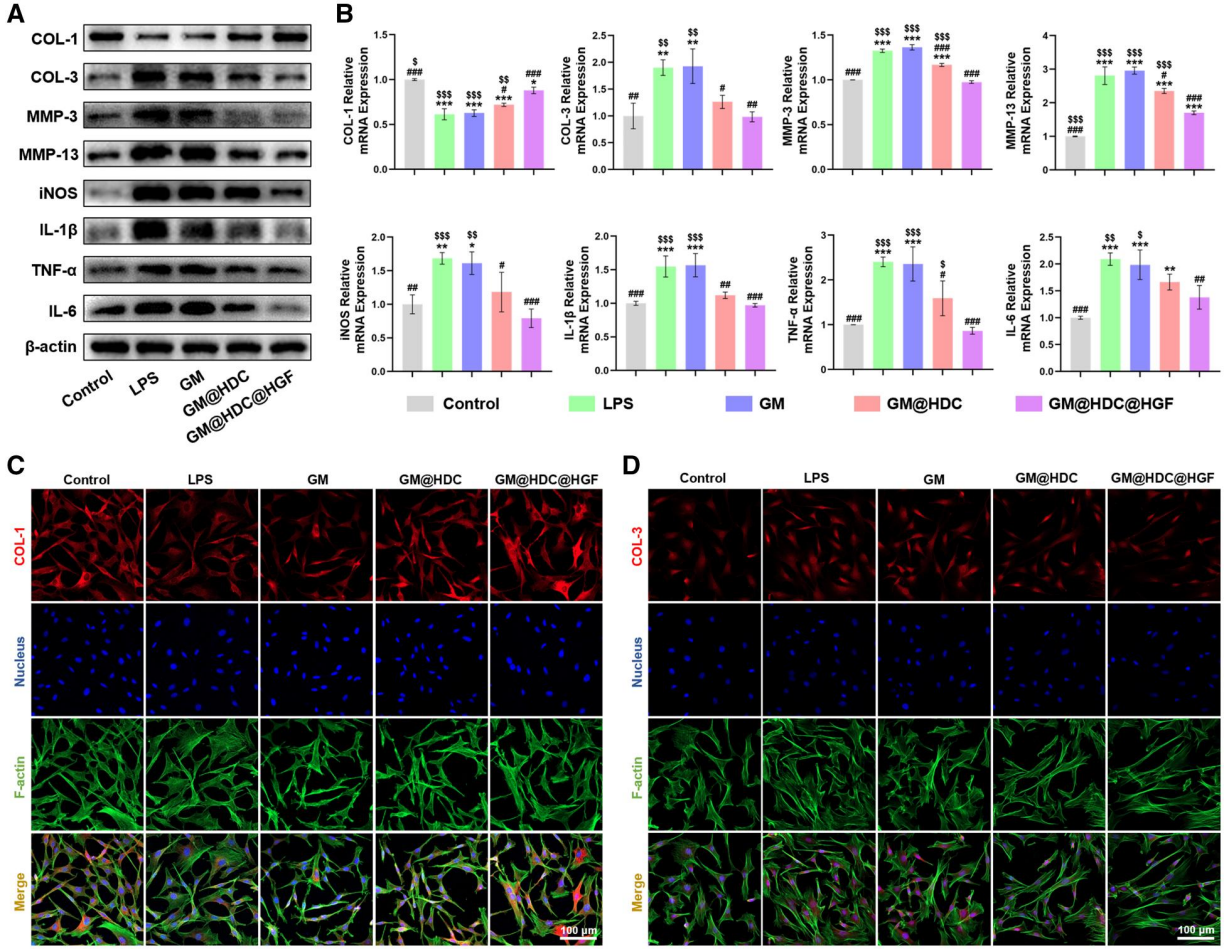
*In vitro* anti-inflammation property of microspheres. (**A**) Western blot of inflammatory factors, ECM degrading enzymes, and ECM proteins. (**B**) mRNA expression level of pro-inflammatory cytokines, ECM degrading enzymes, and ECM proteins. (**C, D**) Representative fluorescence images showing the protein expression level of COL-1 and COL-3 (*n* = 3, */**/***, #/##/### and $/$$/$$$ indicated *P *<* *0.05/*P *<* *0.01/*P *<* *0.001 compared to the control, LPS and GM@HDC@HGF groups, respectively).

Inflammation, which changes the local microenvironment of affected tendons and causes secretion of pro-inflammatory cytokines, plays a key role in the onset of tendinopathy [48]. Excessive inflammatory mediators lead to an imbalance between MMPs and their endogenous inhibitors, adversely changing the microstructure and composition of tendon ECM [8]. Normal tendon matrix is composed of regularly arranged COL-1 and other matrix-related proteins, while the deposition of irregularly arranged COL-3 is significantly increased in tendinopathy, resulting in reduced tendon biomechanical strength. The imbalance of the collagen ratio (COL-3/COL-1) is a characteristic change indicating tendon disease [12]. To detect the inflammation regulation properties of functionalized microspheres *in vitro*, each group was co-cultured with tenocytes induced by LPS. As shown in Figure 5A and Supplementary Figure S5A, Western blot results indicated that LPS-induced tenocytes significantly expressed pro-inflammatory cytokines iNOS, IL-1β, TNF-α, and IL-6, upregulated the protein expression of MMP-3 and MMP-13 and increased the proportion of COL-3/COL-1. Intriguingly, after co-cultured with GM@HDC and GM@HDC@HGF, the expression of inflammation and matrix-related proteins were reversed to some extent. It is worth noting that the GM@HDC@HGF group exhibited better improvement than the GM@HDC group. In addition, mRNA levels were measured accordingly and shared similar trends with protein levels (Figure 5B). These results indicated that HGF and HDC had a synergistic effect in reducing inflammation and improving matrix expression of tenocytes. Immunofluorescence staining analysis of COL-1 and COL-3 was further conducted (Figure 5C and D). The fluorescence intensities of COL-1 and COL-3 were reserved following co-culture with the functionalized microspheres, and the proportion of COL-3/COL-1 tended to normal (Supplementary Figure S5B). The above results suggested that GM@HDC@HGF could effectively control inflammation levels, ameliorate the imbalance of ECM anabolism and catabolism of tenocytes.

### *In vivo* therapeutic effect of AT

**Figure 6.**
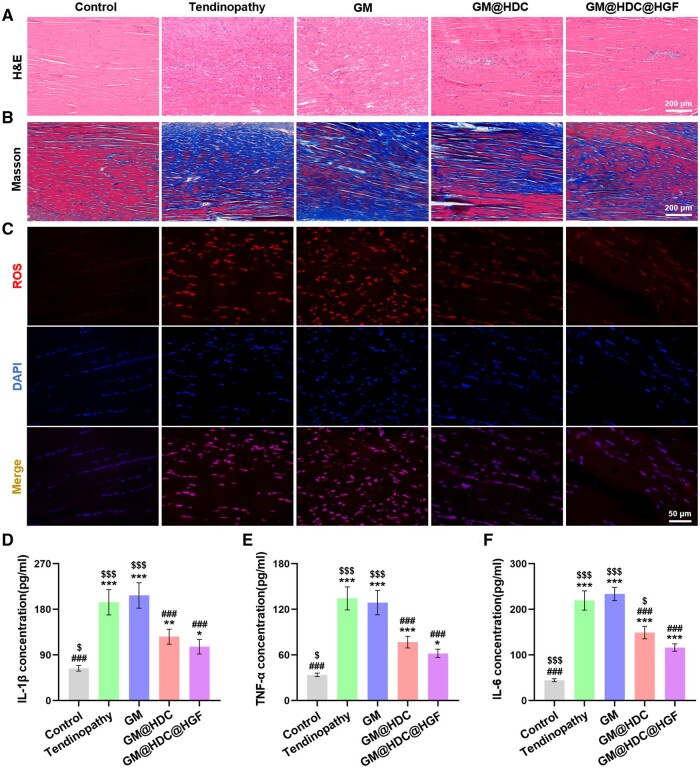
*In vivo* effect of microspheres on inflammation and oxidative stress. (**A**) Representative sections for H&E staining, (**B**) Masson staining, and (**C**) ROS staining of Achilles tendons of rats at 1 week. (**D–F**) Expression of pro-inflammatory cytokines in serum of rats at 1 week (*n* = 4, */**/***, #/##/### and $/$$/$$$ indicated *P *<* *0.05/*P *<* *0.01/*P *<* *0.001 compared to the control, tendinopathy and GM@HDC@HGF groups, respectively).

Rat AT models were established by injection of type I collagenase (Supplementary Figure S6). The acute inflammation stage of tendinopathy lasts about 2 weeks, and then gradually changes to the chronic degeneration stage [49]. One week after administration with the functionalized microspheres, half of the rats were sacrificed to study the inflammation performance. H&E and Masson staining showed that the ECM arrangement of tendons tended to be disordered, except for the control group (Figure 6A and B). In the tendinopathy and GM groups, inflammatory infiltration was significant and collagen degradation was more complete. There was partial collagen degradation in the GM@HDC and GM@HDC@HGF groups, while the degree of inflammatory infiltration was low and fewer capillary clusters were formed. The histological score of tendon sections was evaluated using the classical Bonar score [50]. The GM@HDC and GM@HDC@HGF groups got lower scores than the tendinopathy and GM groups, indicating better outcomes of the two (Supplementary Figure S7A). Frozen section ROS staining was carried out to evaluate the level of oxidative stress of each group (Figure 6C). Almost no ROS positive cells were present in the control group, while a strong ROS staining signal was detected in the tendinopathy and GM groups, accompanied by significant cell proliferations. However, the ROS signal and cell proliferation in the GM@HDC and GM@HDC@HGF groups were significantly lower than those in the former two groups. Supplementary Figure S7B showed the semi-quantitative analysis of the average fluorescence intensity of ROS in each group. It can be seen that GM@HDC and GM@HDC@HGF were equivalent in reducing the high ROS level, suggesting that the inhibition of oxidative stress may be attributed to the HDC component of the material. In addition, the pro-inflammatory cytokines in the serum of rats in each group were detected (Figure 6D–F). Compared with the tendinopathy and GM groups, the IL-1β, TNF-α, and IL-6 levels of the GM@HDC group decreased by approximately 50%, showing no significant difference with GM@HDC@HGF group. These results suggested that functionalized microspheres can effectively alleviate the levels of oxidative stress and inflammation of AT *in vivo*.

**Figure 7.**
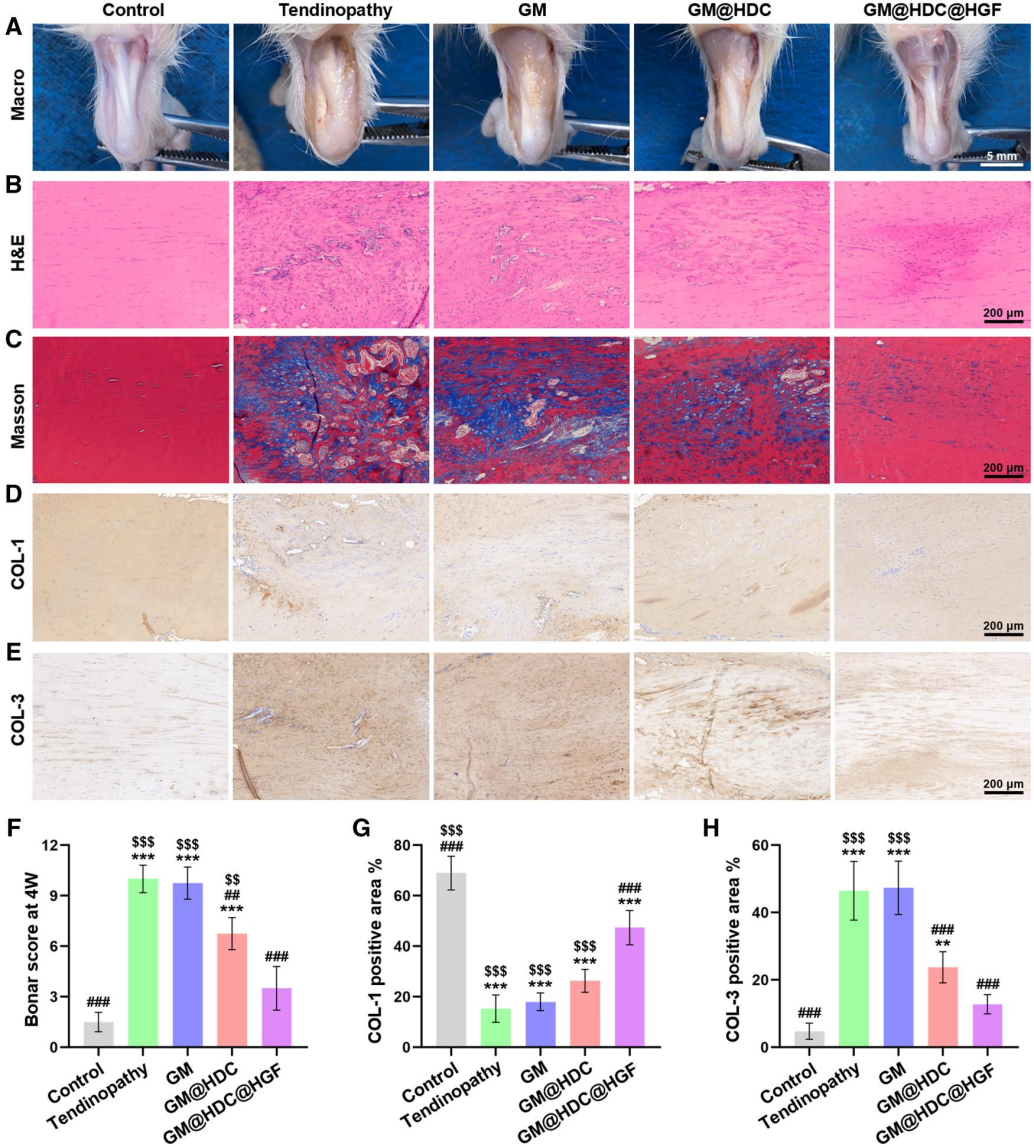
*In vivo* at regeneration with microspheres treatment. (**A**) Macro-picture images of Achille tendons of rats at 4 weeks. (**B**) H&E staining. (**C**) Masson staining. (**D, E**) Immunohistochemistry staining of COL-1 and COL-3. (**F**) Bonar score at 4 weeks. (**G, H**) The proportions of COL-1 and COL-3 positive areas (*n* = 4, */**/***, #/##/### and $/$$/$$$ indicated *P *<* *0.05/*P *<* *0.01/*P *<* *0.001 compared to the control, tendinopathy and GM@HDC@HGF groups, respectively).

After treatment for 4 weeks, the remaining rats were sacrificed to evaluate the regeneration and repair of tendinopathy by the functionalized microspheres. Figure 7A showed the Achille tendon appearance of each group. Macroscopically, tendons in the tendinopathy and GM groups were thicker than those in the control group and were covered by yellowish inflammatory tissue. After treatment with the functionalized microspheres, the transverse width and inflammatory area of tendons in the two groups decreased to some degrees. In the tendinopathy and GM groups, H&E and Masson staining showed disordered arrangement of collagen fibers, chondroid changes of tenocytes, accompanied by cell proliferation and vascularization (Figure 7B and C). After GM@HDC or GM@HDC@HGF treatment, the disorders of tendon tissues turned for the better. Some chondroid cells were still present in the GM@HDC group, while virtually no such cells were observed in the GM@HDC@HGF group. This may be due to the sustained release of HGF, which contributed to the ECM remodeling of tendons. Bonar score showed that GM@HDC@HGF had the most significant effect on AT repairing (Figure 7F). Immunohistochemical staining of COL-1 and COL-3 was performed to further illustrate the therapeutic effect (Figure 7D and E). In the tendinopathy and GM groups, the expression of COL-3 significantly increased while the expression of COL-1 significantly decreased, and the ratio of the two deviated from the normal trend. After treatment of functionalized microspheres, the ratio showed a trend comparable to that of normal tendon. We measured the proportion of positive regions of two kinds of collagen (Figure 7G and H). The results showed that compared with GM@HDC, GM@HDC@HGF were more effective in repairing collagen metabolism disorder. There was no significant difference in the expression of COL-1 between the GM@HDC group and the tendinopathy group. In addition, the tissue proteins of each group were extracted and analyzed for the expression of matrix-related components (COL-1, COL-3, MMP-3, MMP-13) (Supplementary Figure S8). The results were similar to the corresponding *in vitro* findings. In summary, *in situ* injection of GM@HDC@HGF could effectively regulate oxidative stress and inflammation in AT, improve ECM remodeling, and prevent tendon degeneration. However, the pathogenesis of AT is a complex process related to multiple factors. It is necessary to conduct in-depth studies to clarify the specific molecular mechanism of AT and related therapeutic targets.

## Conclusions

In this study, injectable monodisperse hydrogel microspheres were prepared using microfluidic technology and HDC was grafted to the microspheres by biomimetic modification. HGF was further loaded onto GM@HDC by immersed adsorption. GM@HDC@HGF with synergistic antioxidant and anti-inflammatory activities were fabricated for *in situ* treatment of tendinopathy. The HDC modification enhanced the drug loading, degradation, and adhesion of the microspheres. *In vitro* and *in vivo* experiments demonstrated that GM@HDC@HGF could effectively reduce the level of oxidative stress, inhibit the expression of pro-inflammatory cytokines, improve ECM remodeling, and prevent tendon degeneration. We believe that the functionalized microspheres with antioxidant and anti-inflammatory effects are universal and suitable for minimally invasive treatment of a variety of chronic degenerative diseases.

## Supplementary Material

rbae007_Supplementary_Data

## References

[1] Mittal M , Siddiqui MR, Tran K, Reddy SP, Malik AB. Reactive oxygen species in inflammation and tissue injury. Antioxid Redox Signal 2014;20:1126–67. 23991888 10.1089/ars.2012.5149 PMC3929010

[2] Blaser H , Dostert C, Mak TW, Brenner D. TNF and ROS crosstalk in inflammation. Trends Cell Biol 2016;26:249–61. 26791157 10.1016/j.tcb.2015.12.002

[3] McGarry T , Biniecka M, Veale DJ, Fearon U. Hypoxia, oxidative stress and inflammation. Free Radic Biol Med 2018;125:15–24. 29601945 10.1016/j.freeradbiomed.2018.03.042

[4] Martin P , Leibovich SJ. Inflammatory cells during wound repair: the good, the bad and the ugly. Trends Cell Biol 2005;15:599–607. 16202600 10.1016/j.tcb.2005.09.002

[5] Apel K , Hirt H. Reactive oxygen species: metabolism, oxidative stress, and signal transduction. Annu Rev Plant Biol 2004;55:373–99. 15377225 10.1146/annurev.arplant.55.031903.141701

[6] Marchev AS , Dimitrova PA, Burns AJ, Kostov RV, Dinkova-Kostova AT, Georgiev MI. Oxidative stress and chronic inflammation in osteoarthritis: can NRF2 counteract these partners in crime? Ann N Y Acad Sci 2017;1401:114–35. 28662306 10.1111/nyas.13407

[7] Lepetsos P , Papavassiliou KA, Papavassiliou AG. Redox and NF-kappaB signaling in osteoarthritis. Free Radic Biol Med 2019;132:90–100. 30236789 10.1016/j.freeradbiomed.2018.09.025

[8] Tu H , Li YL. Inflammation balance in skeletal muscle damage and repair. Front Immunol 2023;14:1133355. 36776867 10.3389/fimmu.2023.1133355 PMC9909416

[9] Millar NL , Silbernagel KG, Thorborg K, Kirwan PD, Galatz LM, Abrams GD, Murrell GAC, McInnes IB, Rodeo SA. Tendinopathy. Nat Rev Dis Primers 2021;7:1. 33414454 10.1038/s41572-020-00234-1

[10] Scott A , Squier K, Alfredson H, Bahr R, Cook JL, Coombes B, de Vos RJ, Fu SN, Grimaldi A, Lewis JS, Maffulli N, Magnusson SP, Malliaras P, Mc Auliffe S, Oei EHG, Purdam CR, Rees JD, Rio EK, Gravare Silbernagel K, Speed C, Weir A, Wolf JM, Akker-Scheek IVD, Vicenzino BT, Zwerver J. ICON 2019: international scientific tendinopathy symposium consensus: clinical terminology. Br J Sports Med 2020;54:260–2. 31399426 10.1136/bjsports-2019-100885

[11] Millar NL , Murrell GA, McInnes IB. Inflammatory mechanisms in tendinopathy—towards translation. Nat Rev Rheumatol 2017;13:110–22. 28119539 10.1038/nrrheum.2016.213

[12] Magnusson SP , Langberg H, Kjaer M. The pathogenesis of tendinopathy: balancing the response to loading. Nat Rev Rheumatol 2010;6:262–8. 20308995 10.1038/nrrheum.2010.43

[13] Lui PPY , Zhang X, Yao S, Sun H, Huang C. Roles of oxidative stress in acute tendon injury and degenerative tendinopathy – a target for intervention. Int J Mol Sci 2022;23:3571. 35408931 10.3390/ijms23073571 PMC8998577

[14] Lipman K , Wang C, Ting K, Soo C, Zheng Z. Tendinopathy: injury, repair, and current exploration. Drug Des Devel Ther 2018;12:591–603. 10.2147/DDDT.S154660 PMC5865563 29593382

[15] Mellor R , Bennell K, Grimaldi A, Nicolson P, Kasza J, Hodges P, Wajswelner H, Vicenzino B. Education plus exercise versus corticosteroid injection use versus a wait and see approach on global outcome and pain from gluteal tendinopathy: prospective, single blinded, randomised clinical trial. BMJ 2018;361:k1662. 29720374 10.1136/bmj.k1662 PMC5930290

[16] Bao D , Sun J, Gong M, Shi J, Qin B, Deng K, Liu G, Zeng S, Xiang Z, Fu S. Combination of graphene oxide and platelet-rich plasma improves tendon-bone healing in a rabbit model of supraspinatus tendon reconstruction. Regen Biomater 2021;8:rbab045. 34484806 10.1093/rb/rbab045 PMC8411035

[17] Loiacono C , Palermi S, Massa B, Belviso I, Romano V, Gregorio AD, Sirico F, Sacco AM. Tendinopathy: pathophysiology, therapeutic options, and role of nutraceutics. A narrative literature review. Medicina (Kaunas) 2019;55:447. 31394838 10.3390/medicina55080447 PMC6723894

[18] Appetecchia F , Consalvi S, Berrino E, Gallorini M, Granese A, Campestre C, Carradori S, Biava M, Poce G. A novel class of dual-acting DCH-CORMs counteracts oxidative stress-induced inflammation in human primary tenocytes. Antioxidants (Basel) 2021;10:1828. 34829699 10.3390/antiox10111828 PMC8614895

[19] Semis HS , Gur C, Ileriturk M, Kandemir FM, Kaynar O. Evaluation of therapeutic effects of quercetin against Achilles tendinopathy in rats via oxidative stress, inflammation, apoptosis, autophagy, and metalloproteinases. Am J Sports Med 2022;50:486–98. 34908488 10.1177/03635465211059821

[20] Aicale R , Bisaccia RD, Oliviero A, Oliva F, Maffulli N. Current pharmacological approaches to the treatment of tendinopathy. Expert Opin Pharmacother 2020;21:1467–77. 32511031 10.1080/14656566.2020.1763306

[21] Brinks A , Koes BW, Volkers AC, Verhaar JA, Bierma-Zeinstra SM. Adverse effects of extra-articular corticosteroid injections: a systematic review. BMC Musculoskelet Disord 2010;11:206. 20836867 10.1186/1471-2474-11-206 PMC2945953

[22] Chahla J , Cinque ME, Piuzzi NS, Mannava S, Geeslin AG, Murray IR, Dornan GJ, Muschler GF, LaPrade RF. A call for standardization in platelet-rich plasma preparation protocols and composition reporting: a systematic review of the clinical orthopaedic literature. J Bone Joint Surg Am 2017;99:1769–79. 29040132 10.2106/JBJS.16.01374

[23] Beldjilali-Labro M , Garcia Garcia A, Farhat F, Bedoui F, Grosset JF, Dufresne M, Legallais C. Biomaterials in tendon and skeletal muscle tissue engineering: current trends and challenges. Materials (Basel) 2018;11:1116. 29966303 10.3390/ma11071116 PMC6073924

[24] Tang Y , Wang Z, Xiang L, Zhao Z, Cui W. Functional biomaterials for tendon/ligament repair and regeneration. Regen Biomater 2022;9:rbac062. 36176715 10.1093/rb/rbac062 PMC9514853

[25] Freedman BR , Mooney DJ, Weber E. Advances toward transformative therapies for tendon diseases. Sci Transl Med 2022;14:eabl8814. 36070365 10.1126/scitranslmed.abl8814 PMC11041812

[26] Martins C , Sousa F, Araujo F, Sarmento B. Functionalizing PLGA and PLGA derivatives for drug delivery and tissue regeneration applications. Adv Healthc Mater 2018;7:1701035. 10.1002/adhm.201701035 29171928

[27] Yao S , Liu H, Yu S, Li Y, Wang X, Wang L. Drug-nanoencapsulated PLGA microspheres prepared by emulsion electrospray with controlled release behavior. Regen Biomater 2016;3:309–17. 27699061 10.1093/rb/rbw033 PMC5043157

[28] Lih E , Park W, Park KW, Chun SY, Kim H, Joung YK, Kwon TG, Hubbell JA, Han DK. A bioinspired scaffold with anti-inflammatory magnesium hydroxide and decellularized extracellular matrix for renal tissue regeneration. ACS Cent Sci 2019;5:458–67. 30937373 10.1021/acscentsci.8b00812 PMC6439446

[29] Li J , Chen G, Xu X, Abdou P, Jiang Q, Shi D, Gu Z. Advances of injectable hydrogel-based scaffolds for cartilage regeneration. Regen Biomater 2019;6:129–40. 31198581 10.1093/rb/rbz022 PMC6547311

[30] Garving C , Jakob S, Bauer I, Nadjar R, Brunner UH. Impingement syndrome of the shoulder. Dtsch Arztebl Int 2017;114:765–76. 29202926 10.3238/arztebl.2017.0765 PMC5729225

[31] Li W , Yang X, Lai P, Shang L. Bio‐inspired adhesive hydrogel for biomedicine—principles and design strategies. Smart Med 2022;1:e20220024. 10.1002/SMMD.20220024 PMC11235927 39188733

[32] Tonomura H , Nagae M, Takatori R, Ishibashi H, Itsuji T, Takahashi K. The potential role of hepatocyte growth factor in degenerative disorders of the synovial joint and spine. Int J Mol Sci 2020;21:8717. 33218127 10.3390/ijms21228717 PMC7698933

[33] Han P , Cui Q, Lu W, Yang S, Shi M, Li Z, Gao P, Xu B, Li Z. Hepatocyte growth factor plays a dual role in tendon-derived stem cell proliferation, migration, and differentiation. J Cell Physiol 2019;234:17382–91. 30807656 10.1002/jcp.28360

[34] Zhang J , Middleton KK, Fu FH, Im HJ, Wang JH. HGF mediates the anti-inflammatory effects of PRP on injured tendons. PLoS One 2013;8:e67303. 23840657 10.1371/journal.pone.0067303 PMC3696073

[35] Zhou H , Casas-Finet JR, Heath Coats R, Kaufman JD, Stahl SJ, Wingfield PT, Rubin JS, Bottaro DP, Byrd RA. Identification and dynamics of a heparin-binding site in hepatocyte growth factor. Biochemistry 1999;38:14793–802. 10555961 10.1021/bi9908641

[36] Ikegami Y , Mizumachi H, Yoshida K, Ijima H. Heparin-conjugated collagen as a potent growth factor-localizing and stabilizing scaffold for regenerative medicine. Regen Ther 2020;15:236–42. 33426224 10.1016/j.reth.2020.10.002 PMC7770420

[37] Bian J , Cai F, Chen H, Tang Z, Xi K, Tang J, Wu L, Xu Y, Deng L, Gu Y, Cui W, Chen L. Modulation of local overactive inflammation via injectable hydrogel microspheres. Nano Lett 2021;21:2690–8. 33543616 10.1021/acs.nanolett.0c04713

[38] Wu J , Zhu J, Wu Q, An Y, Wang K, Xuan T, Zhang J, Song W, He H, Song L, Zheng J, Xiao J. Mussel-inspired surface immobilization of heparin on magnetic nanoparticles for enhanced wound repair via sustained release of a growth factor and M2 macrophage polarization. ACS Appl Mater Interfaces 2021;13:2230–44. 33403850 10.1021/acsami.0c18388

[39] Li Y , Yang L, Hou Y, Zhang Z, Chen M, Wang M, Liu J, Wang J, Zhao Z, Xie C, Lu X. Polydopamine-mediated graphene oxide and nanohydroxyapatite-incorporated conductive scaffold with an immunomodulatory ability accelerates periodontal bone regeneration in diabetes. Bioact Mater 2022;18:213–27. 35387166 10.1016/j.bioactmat.2022.03.021 PMC8961429

[40] Piao Y , You H, Xu T, Bei H-P, Piwko IZ, Kwan YY, Zhao X. Biomedical applications of gelatin methacryloyl hydrogels. Eng Regen 2021;2:47–56.

[41] Lei Y , Zhang Q, Kuang G, Wang X, Fan Q, Ye F. Functional biomaterials for osteoarthritis treatment: from research to application. Smart Med 2022;1:e20220014. 10.1002/SMMD.20220014 PMC11235767 39188730

[42] Zhuge W , Liu H, Wang W, Wang J. Microfluidic bioscaffolds for regenerative engineering. Eng Regen 2022;3:110–20.

[43] Zhao X , Lang Q, Yildirimer L, Lin ZY, Cui W, Annabi N, Ng KW, Dokmeci MR, Ghaemmaghami AM, Khademhosseini A. Photocrosslinkable gelatin hydrogel for epidermal tissue engineering. Adv Healthc Mater 2016;5:108–18. 25880725 10.1002/adhm.201500005 PMC4608855

[44] Asha AB , Chen Y, Narain R. Bioinspired dopamine and zwitterionic polymers for non-fouling surface engineering. Chem Soc Rev 2021;50:11668–83. 34477190 10.1039/d1cs00658d

[45] Wu F , Nerlich M, Docheva D. Tendon injuries: basic science and new repair proposals. EFORT Open Rev 2017;2:332–42. 28828182 10.1302/2058-5241.2.160075 PMC5549180

[46] Qi J , Wang Y, Chen L, Chen L, Wen F, Huang L, Zhang RP, Li CH. 3D-printed porous functional composite scaffolds with polydopamine decoration for bone regeneration. Regen Biomater 2023;10:rbad062. 37520855 10.1093/rb/rbad062 PMC10374492

[47] Hussain T , Tan B, Yin Y, Blachier F, Tossou MC, Rahu N. Oxidative stress and inflammation: what polyphenols can do for us? Oxid Med Cell Longev 2016;2016:7432797. 27738491 10.1155/2016/7432797 PMC5055983

[48] Tang C , Chen Y, Huang J, Zhao K, Chen X, Yin Z, Heng BC, Chen W, Shen W. The roles of inflammatory mediators and immunocytes in tendinopathy. J Orthop Translat 2018;14:23–33. 30035030 10.1016/j.jot.2018.03.003 PMC6034108

[49] Perucca Orfei C , Lovati AB, Vigano M, Stanco D, Bottagisio M, Di Giancamillo A, Setti S, de Girolamo L. Dose-related and time-dependent development of collagenase-induced tendinopathy in rats. PLoS One 2016;11:e0161590. 27548063 10.1371/journal.pone.0161590 PMC4993508

[50] Maffulli N , Longo UG, Franceschi F, Rabitti C, Denaro V. Movin and Bonar scores assess the same characteristics of tendon histology. Clin Orthop Relat Res 2008;466:1605–11. 18437501 10.1007/s11999-008-0261-0 PMC2505245

