# Supplementary material for: Antioxidant and anti-inflammatory injectable hydrogel microspheres for in situ treatment of tendinopathy
Source: Regen Biomater. 2024 Jan 30;11:rbae007. doi: 10.1093/rb/rbae007 (PMC10898336; doi:10.1093/rb/rbae007)
Supplement: rbae007_Supplementary_Data [file rbae007_supplementary_data.zip › Final Supplementary File.docx]

**Antioxidant and anti-inflammatory injectable hydrogel microspheres for in situ treatment of tendinopathy**

Qibin Han ^a,b,†^, Lang Bai ^a,b,†^, Yinhua Qian ^c,†^, Xiaoyu Zhang ^a,b^, Juan Wang ^d^, Jing Zhou ^a,b^, Wenguo Cui ^d,*^, Yuefeng Hao ^a,b,*^, Xing Yang ^a,b,*^

^a^Department of Orthopedics, The Affiliated Suzhou Hospital of Nanjing Medical University, 242 Guangji Road, Suzhou 215008, P. R. China.

^b^Gusu School, Nanjing Medical University, 458 Shizi Road, Suzhou 215006, P. R. China.

^c^Department of Orthopedics, Kunshan Hospital of Traditional Chinese Medicine, 388 Zuchongzhi South Road, Suzhou 215300, P. R. China.

^d^Department of Orthopaedics, Shanghai Key Laboratory for Prevention and Treatment of Bone and Joint Diseases, Shanghai Institute of Traumatology and Orthopaedics, Ruijin Hospital, Shanghai Jiao Tong University School of Medicine, 197 Ruijin 2nd Road, Shanghai 200025, P. R. China.

^†^These authors contributed equally to this work.

*Corresponding authors:

Wenguo Cui: wgcui80@hotmail.com

Yuefeng Hao: 13913109339@163.com

Xing Yang: xingyangsz@126.com


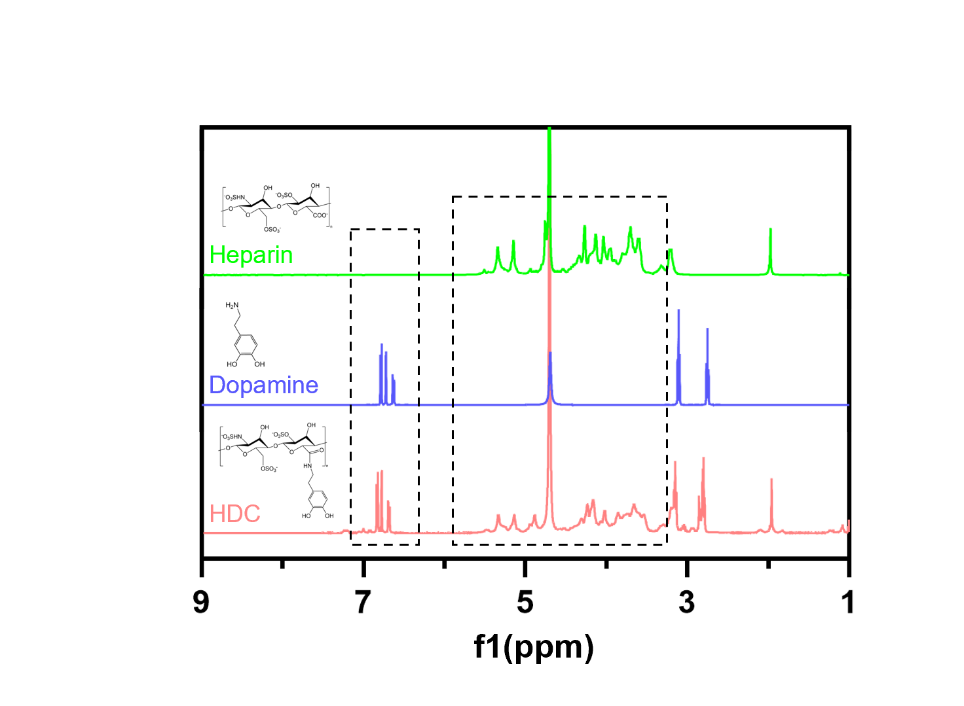


**Supplementary Figure S1.** ^1^H NMR of heparin, dopamine and HDC.


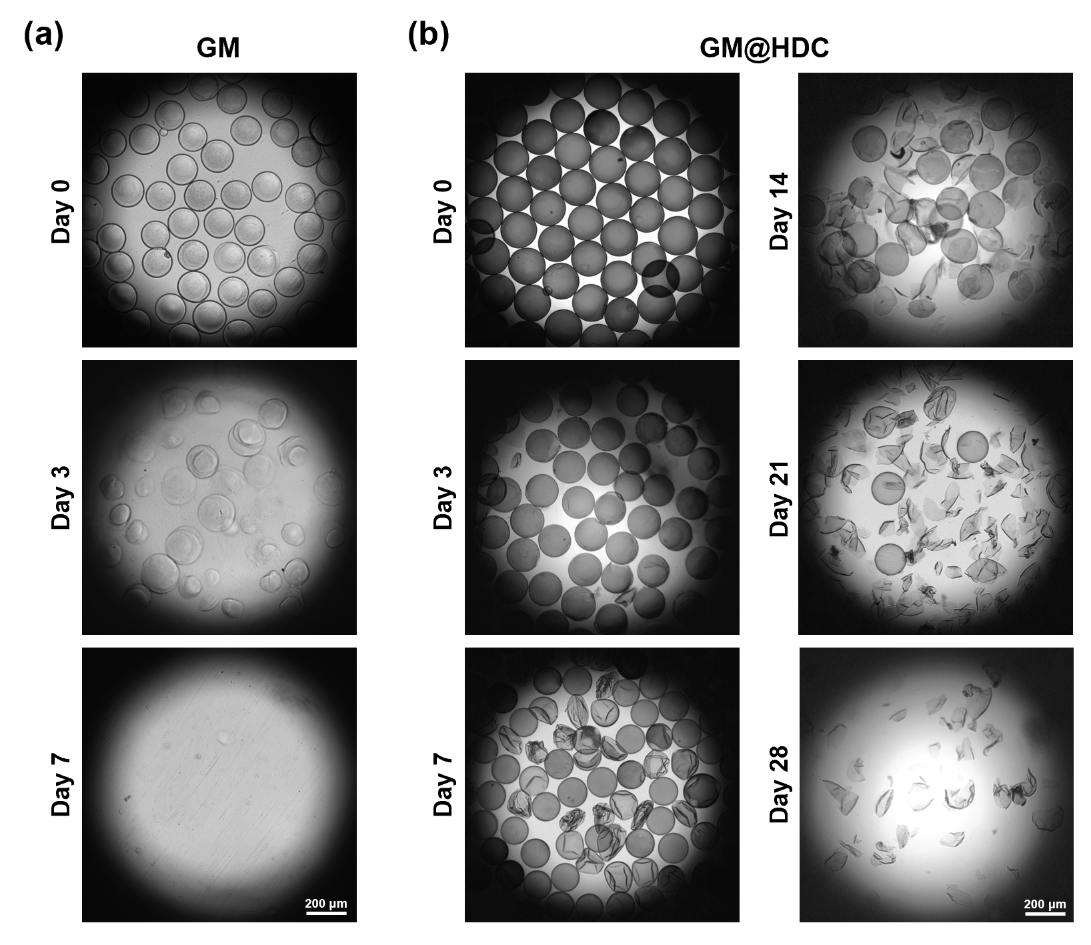


**Supplementary Figure S2.** Representative images of a) GM and b) GM@HDC incubated with collagenase at different time points.


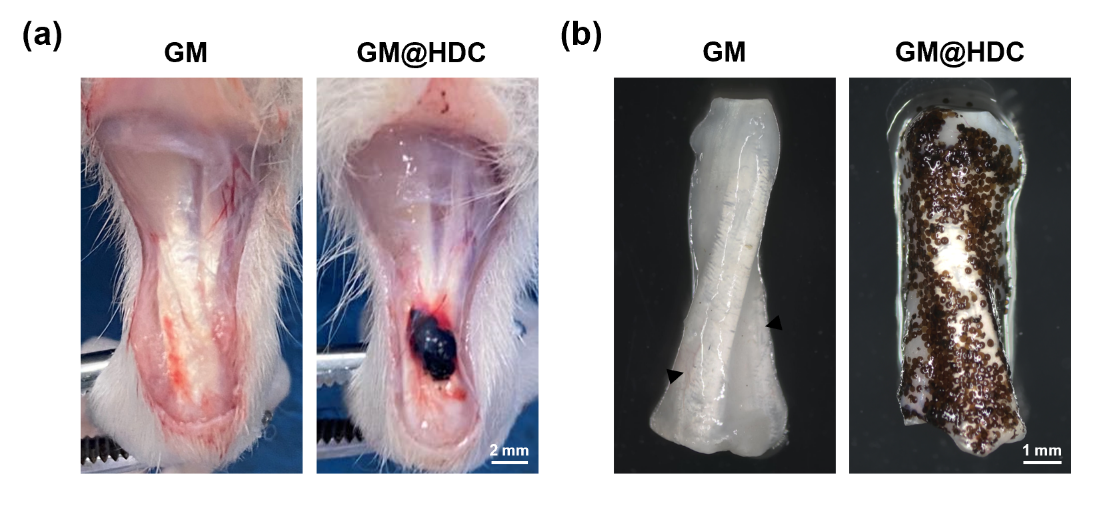


**Supplementary Figure S3.** Characterization of adhesion property of GM and GM@HDC. a) Distribution of GM or GM@HDC injected in situ for 24 h. b) Images of isolated rat Achille tendons immersed with GM or GM@HDC for 30 min.


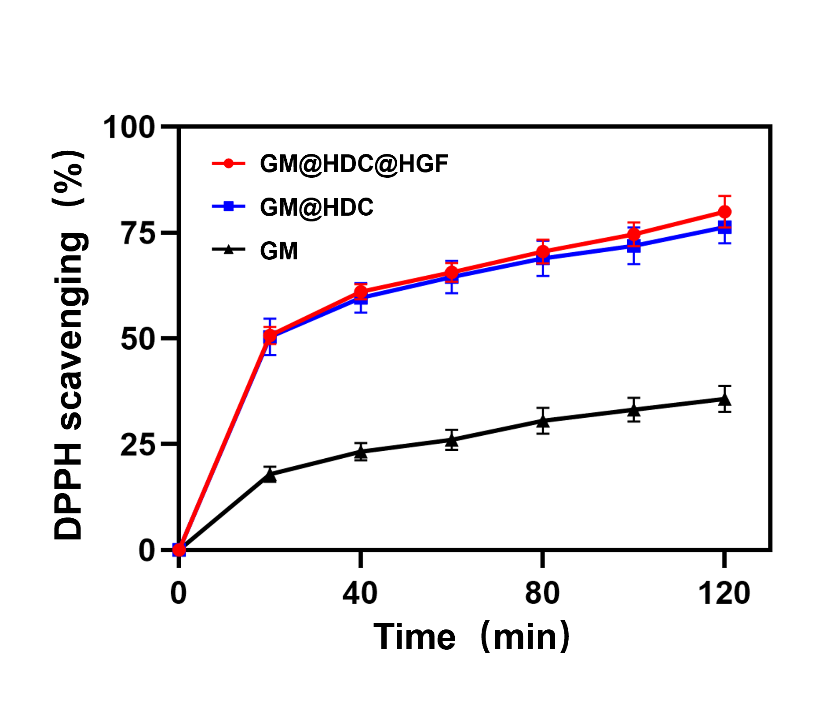


**Supplementary Figure S4.** DPPH scavenging efficiency of GM, GM@HDC and GM@HDC@HGF. (n = 3).


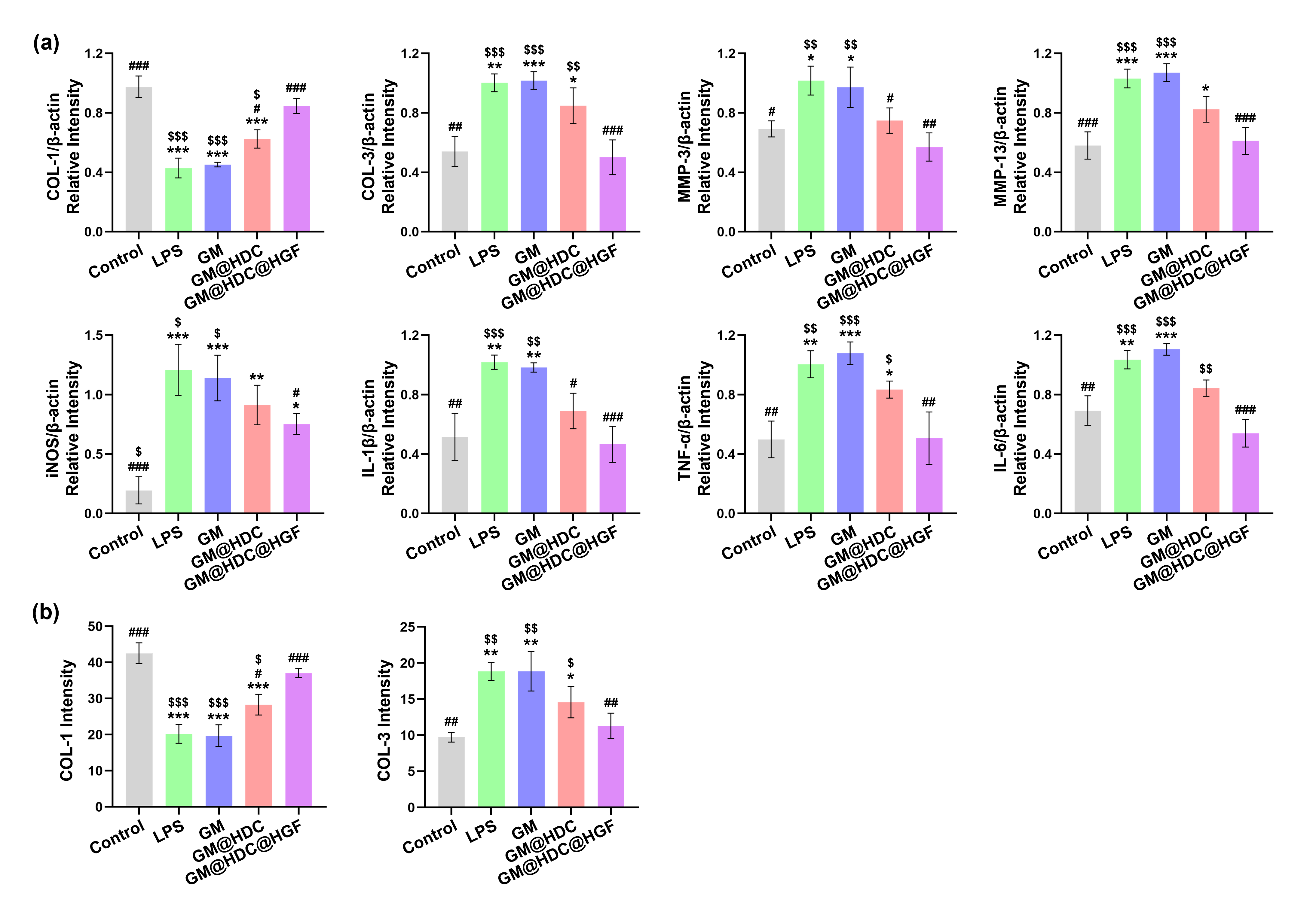


**Supplementary Figure S5.** a) Semi-quantitative analysis of expression of pro-inflammatory cytokines, ECM degrading enzymes, and ECM proteins. b) fluorescence intensity of COL-1 and COL-3. (n = 3, */**/***, #/##/### and $/$$/$$$ indicated *p* < 0.05/*p* < 0.01/*p* < 0.001 compared to the Control, LPS and GM@HDC@HGF groups, respectively).


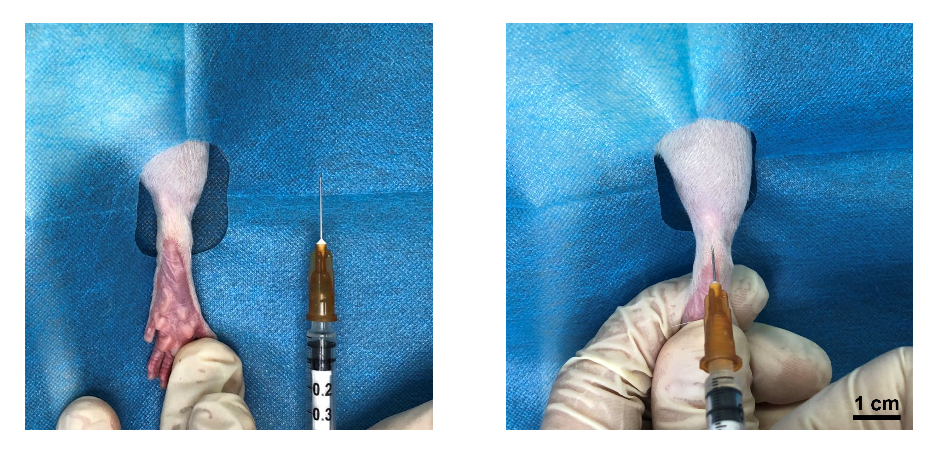


**Supplementary Figure S6.** Establishment of the rat AT model.


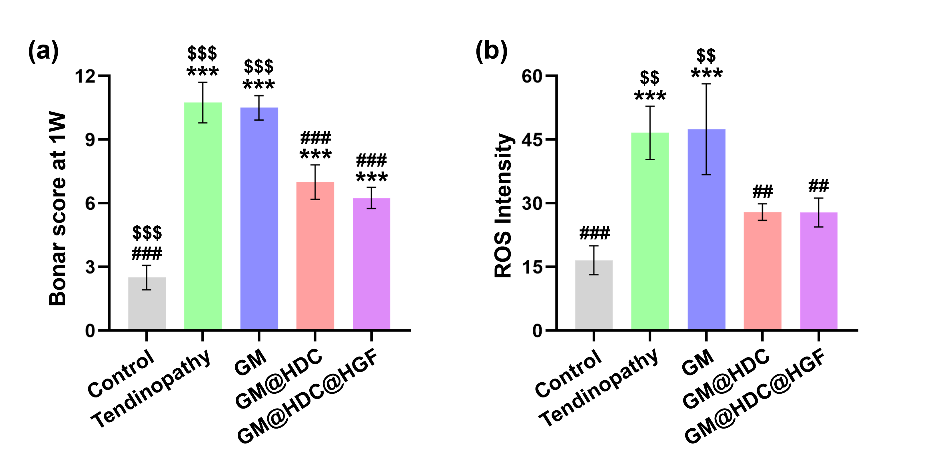


**Supplementary Figure S7.** a) Bonar score at 1 week and b) semi-quantitative analysis of ROS intensity. (n = 4, */**/***, #/##/### and $/$$/$$$ indicated *p* < 0.05/*p* < 0.01/*p* < 0.001 compared to the Control, Tendinopathy and GM@HDC@HGF groups, respectively).


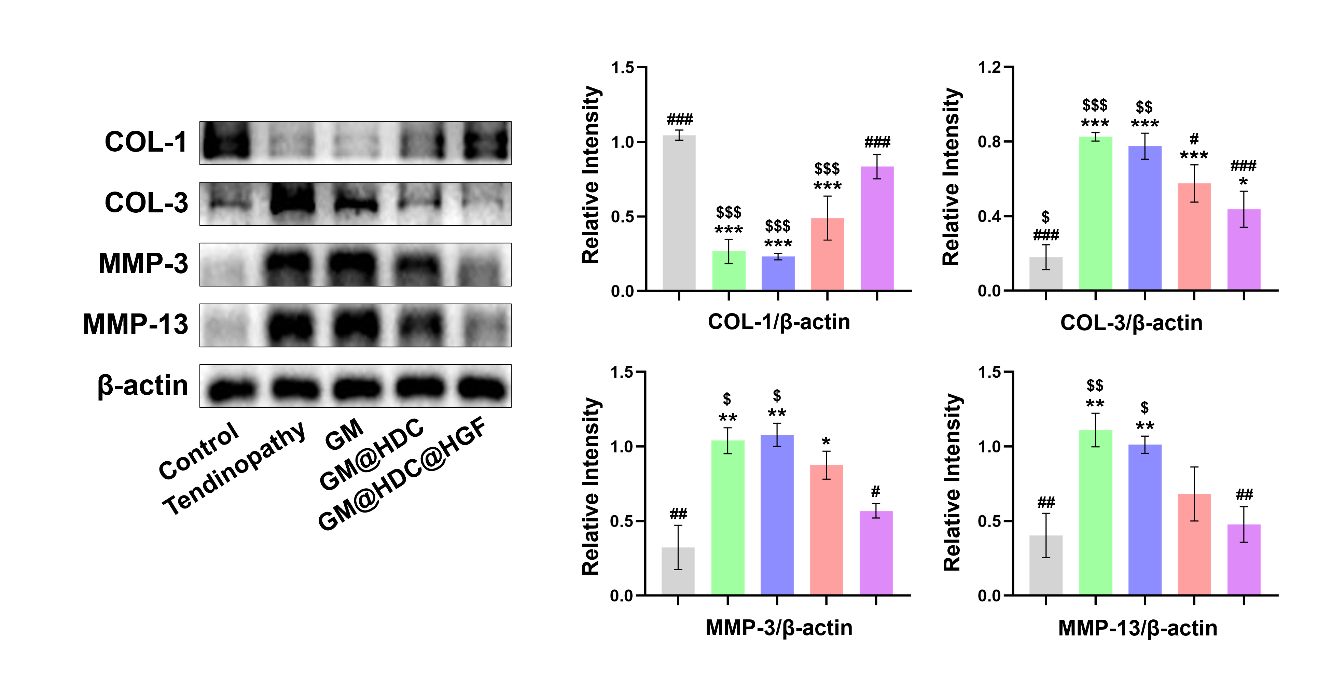


**Supplementary Figure S8.** a) Western blot and b) semi-quantitative analysis of matrix-related proteins of rat Achilles tendon tissues at 4 weeks. (n = 3, */**/***, #/##/### and $/$$/$$$ indicated *p* < 0.05/*p* < 0.01/*p* < 0.001 compared to the Control, Tendinopathy and GM@HDC@HGF groups, respectively).
