# Supplementary material for: Antioxidant and anti-inflammatory injectable hydrogel microspheres for in situ treatment of tendinopathy
Source: Regen Biomater. 2024 Jan 30;11:rbae007. doi: 10.1093/rb/rbae007 (PMC10898336; doi:10.1093/rb/rbae007)
Supplement: rbae007_Supplementary_Data [file rbae007_supplementary_data.zip › Response letter.docx]

Dear Editor,

We are grateful to the editors and reviewers for their time and comments on our manuscript. We have implemented their comments and suggestions and are submitting a revised version of the manuscript for further consideration in your journal. Our point-by-point responses addressing each of the reviewers’ comments are listed below. We look forward to hearing from you at your earliest convenience. Thank you.

Yours sincerely,

On behalf of the co-authors

Wenguo Cui, Ph.D./ Prof.

**Response to Reviewers’ Comments**

**Reviewer #1:**

In this work, Han et al. designed and fabricated injectable methacrylate gelatin (GelMA) microspheres (GM) loaded with heparin-dopamine conjugate and hepatocyte growth factors.

In general, GM@HDC@HGF enabled to balance the inflammatory microenvironment by inhibiting oxidative stress and inflammation, thereby regulating extracellular matrix metabolism and halting tendon degeneration. The catechol groups on dopamine have adhesion and antioxidant properties, allowing potential attachment at the injured site, and better function synergized with HGF. GM@HDC@HGF injected in situ in rat Achilles tendinopathy models significantly down-regulated oxidative stress and inflammation, and ameliorated ECM degeneration. In conclusion, the multifunctional platform developed presents a promising alternative for the treatment of tendinopathy.

Overall, this work is well-organized and the characterizations in both materials and in vivo/vitro tests are quite comprehensive. Therefore, I believe it can be accepted once some of raised concerns are fully addressed.

1. Many grammar issues exist in the main article, which makes some paragraphs hard to understand. For instance, “Researchers prefer to in situ intervention at the lesion site when designing materials” …

Response: Thank you for your careful review of our study. Sorry for the inconvenience in reading some of the contexts. We have invited a native English-speaker to correct the grammatical errors in the manuscript.

2. What is the yield of this synthesized GelMA, and how about the substitution rate (or grafting rate) of MA on each macromolecular chain?

Response: Thank you very much for your comment. We have measured that 13.25 g GelMA were obtained from 20 g gelatin according to our protocol. The mass loss was mainly due to the consumption during the process. In fact, a large number of microspheres can be fabricated with a small amount of GelMA hydrogel. The grafting rate of MA changes with the ratio of raw materials. We did not characterize the grafting rate of MA of the synthesized GelMA. According to Ref. 41 (PMID: 25880725) in which the same GelMA synthesis protocol was applied, the synthesized GelMA was found to have a degree of MA modification of 75%.

3. The concept of “injectable hydrogels” in this work is quite confusing, because it is not bulk hydrogels with reversible chemical bonding. The overall synthesized sample should be microsphere solutions?

Response: Thank you very much for this question. The phrase “injectable hydrogel” should be seen in combination with the subsequent word “microspheres” as “injectable hydrogel microspheres”. GM@HDC@HGF was a kind of modified microspheres and fabricated based on the GelMA hydrogel. We actually wanted to highlight the concept of “injectable microspheres” instead of “injectable hydrogel”.

4. The modification of catechol groups is based on the EDC/NHS coupling reaction. However, the key steps should be the diffusion of dopamine small molecules. How to control that?

Response: Thank you very much for the comment. It is critical to control the diffusion of dopamine molecules when synthesizing HDC by EDC/HNS method in order to ensure the effectiveness of the reaction and the purity of the products. Firstly, we employ transparent and inert containers to minimize nonspecific adsorption of dopamine to the container surfaces. Secondly, air within the container was replaced by nitrogen to avoid the effect of oxygen. Thirdly, we maintained an appropriate stirring speed to ensure the even distribution of reactants and prevent uneven reactions caused by local concentration differences.

5. Figure 7. In vivo AT regeneration with microspheres treatment (4 weeks). The tendinopathy and GM groups, the expression of COL-3 significantly increased while the expression of COL-1 significantly decreased, and the ratio of the two deviated from the normal trend. Why?

Response: Thank you for your question. COL-1 is a major component in the ECM of normal tendons, whereas COL-3 accounts for only a small proportion. Studies have shown that ECM degeneration is a critical feature of tendinopathy. In the early stage of tendinopathy, COL-3 is produced and acts as a "patch" to protect the injured tendons. During the tendon healing process, aligned COL-1 fibers gradually replace COL-3. The normal tendon healing process can be inhibited by inflammation or repetitive mechanical loading, with insufficient COL-1 to replace the excessive COL-3, ultimately exacerbating tendinopathy. Thus, the ratio of the two collagens is often used to evaluate the prognosis of tendinopathy.

6. Add the scale bar in Figure 7a.

Response: Thank you for your comment. We have added the scale bar in Figure 7a in the revised manuscript. Please see below:

**Reviewer #2:**

Han et al. presented a novel work using dopamine-coated GelMA microspheres loaded with HGF for tendinopathy treatment. While fully characterizing all aspects of the material, the reader is shown the potentially great application value. In my opinion, this work has met the requirements for publication; however, some details should be subject to minor revision.

(1) In the introduction, lines 71 and 95, the authors describe "regeneration of injured tendons", which could be changed given that the study used a non-traumatic animal model.

Response: Thank you very much for your comment. We feel sorry for the inaccurate statement. It is very hard to say that modified microspheres can promote tendon regeneration. Based on our results, a more accurate description would be that modified microspheres inhibit or prevent tendon degeneration. We have replaced the relevant description about “promote regeneration of injured tendons” with “prevent degeneration of affected tendons” in the revised manuscript.

(2) There is no discussion in the manuscript or comparison to other studies (PMID: 32311538, 34921569, 36481504) as to why surface charge attraction was chosen as the loading method for HGF. And why GelMA microspheres were lyophilized? In fact, it seems feasible if HGF is dissolved in the water-phase solution. Are the authors motivated by considerations of drug loading concentration or drug release rate? and could this be discussed appropriately?

Response: Thank you very much for your questions. We chose to load HGF by charge attraction out of the following considerations: 1) As a small molecule substance, HGF is difficult to be efficiently loaded into GelMA microspheres. The loose reticular structure of GelMA makes it easy for HGF to diffuse outward. In contrast, in PMID: 36481504, MMP1, which is also a small molecule substance, was grafted onto a self-assembled peptide, enabling it to be retained by the reticular structure of GelMA. 2) As a growth factor, HGF is readily inactivated by exposure to external stimuli. Suppose that HGF is added into GelMA solution to prepare microspheres, it can be severely influenced by the organic reagents during the subsequent cleaning process of microspheres. 3) As a heparin-binding growth factor, HGF is able to be loaded into specific structural domains of heparin molecule, which better ensures its bioactivity and avoids the drawbacks mentioned above. Our results also demonstrated that GM@HDC has a higher HGF encapsulation rate and longer release profile than GM. In addition, the lyophilized GelMA microspheres have a loose and porous surface, which increases their specific surface area, thus enabling the microspheres to be loaded with more drugs. We have included some relevant content in the “Introduction” section:

In this study, injectable methacrylate gelatin (GelMA) microspheres (GM) functionalized with dopamine and hepatocyte growth factor (HGF) were fabricated to inhibit oxidative stress and inflammation at the pathogenic sites of tendinopathy, regulate the local tendon microenvironment, ameliorate ECM metabolic disorders, and prevent tendon degeneration. The catechol groups in dopamine have excellent adhesion and antioxidant properties and can bind firmly to the surface of GM as well as to the affected tendons after in situ injection, making it available to perform its physiological functions [31]. HGF is a multifunctional growth factor initially found in liver with anti-inflammatory and matrix-regulation effects [32]. Studies have shown that HGF can promote proliferation and migration of tendon-derived stem cells and mitigate inflammation in injured tendons [33, 34]. Additionally, as a heparin-binding growth factor, HGF can form stable bonds with the heparin domain to avoid inactivation by external interference [35, 36]. Taking advantage of this, dopamine was combined with heparin via an amidation reaction to form the heparin-dopamine conjugate (HDC), which was used as a bridge to efficiently bind HGF to GM. In addition, the porous properties of GM ensured a high drug loading rate.

(3) The in vitro anti-inflammation properties were mainly used with tenocytes, and the role of immune cells in this could be discussed further.

Response: Thank you for your kindly suggestion. It is important and meaningful to further explore the role of immune cells because studies have shown that various immune cell subtypes and inflammatory mediators have a key role in the initiation and progression of tendinopathy. The various immune cells and activated tenocytes produce numerous inflammatory cytokines and chemokines that initiate and augment the adverse alterations in tendon microstructure and composition. It is a limitation of our study that we only focused on the phenotypes of tenocytes. We will try to discuss the role of immune cells in future studies.

(4) Mean ± standard deviation should be used when describing detailed values.

Response: Thank you very much for your helpful observation. We have replaced detailed values with mean ± standard deviation in the revised manuscript. Sorry for the mistakes.

(5) Scale bars should be added to fig. S3, fig. S6, and fig. 7a.

Response: Thank you for your comment. We have added the scale bar to the corresponding figures in the revised manuscript. Please see below:

Fig. S3

Fig. S6

Fig. 7a

**Reviewer #3:**

1. The live/dead staining images (Figure 3a) indicated the GM@HDC group had more cells and better spread, whereas Figure 3c suggested the GM@HDC@HGF group displayed the highest cell vitality. However, these findings contradict each other. Additionally, nearly no dead fluorescence could be observed in all groups, it was well known, even in the control group, there would be dead cells present. To accurately assess cell viability using LIVE/DEAD staining, incorporating DAPI staining is crucial due to the tenocytes' tendency to spread post-inoculating and relying solely on green fluorescence area or intensity results in significant inaccuracies.

Response: Thank you very much for the comment. We apologize for the misplacement of representative fluorescence images. In fact, we utilized cell counting instead of fluorescence area or intensity for cell viability analysis. Red-stained dead cells did exist in some images, and they were not recognized because the red fluorescence intensity was too low. We have uniformly increased the red fluorescence intensity of each image and replaced some representative images so that they can be consistent with the statistical analysis. In addition, it is reasonable to apply nuclear staining at the same time. We will consider combining live/dead staining with DAPI staining in future studies.

2. What are the reasons for choosing a concentration of H_2_O_2_ of 100 μM?

Response: Thank you for your comment. A variety of intervention concentrations have been used to induce oxidative stress injury in tenocytes. 100 μM H_2_O_2_ has been used in many in vitro oxidative stress models, such as chondrocytes and nucleus pulposus cells. According to our pre-tests, excessive concentration of H_2_O_2_ tends to cause tenocytes death, while a lower concentration results in a nonsignificant oxidative injury phenotype. Therefore, we chose 100 μM H_2_O_2_ for further study.

3. In Figure 4b, why is the ROS content in the GM group higher than that in the H_2_O_2_ group

Response: Thank you for your observation. Combining with other images in Figure 4b, it can be seen that the degree of oxidative damage in the H_2_O_2_ and GM groups was close to each other because GelMA microspheres alone do not have antioxidant capacity. In Figure 4b, the proportion of ROS-positive cells in the H_2_O_2_ and GM groups was also very close. Actually, statistical analysis of corresponding repeated experiments showed no significant difference in the proportion of ROS-positive cells between the two groups.

4. Why is LPS used to induce inflammation in in vitro cell experiments? As is well known, LPS is produced by bacteria, and tendinitis is a sterile inflammatory disease.

Response: Thank you very much for your comment. Although many researchers use LPS to induce inflammation in tenocytes, it should be more rational to use pro-inflammatory factors of non-bacterial origin such as TNF-α and IL-1β. The choice of the corresponding in vitro model for tendinopathy has been controversial because collagenase does not work well in cellular experiments. We chose LPS for in vitro experiments because it is considered as a classical substance for inducing inflammation. It is regretful that we did not use a sterile in vitro inflammation model.

5. Why choose 1ug/mL for LPS inflammation induction concentration. What is the bacterial source of LPS?

Response: Thank you for your question. We referred to several studies about tendinopathy among which 1ug/mL LPS was commonly used to establishing in vitro inflammation model (e.g. PMID 29534523 & 30732820). Such concentration of LPS is relatively high and often used to induce inflammation in primary cells, which tend to be less sensitive to stimulus than cell lines.

**Reviewer #4:**

The authors fabricated injective and adherent microspheres based on modified GelMA for efficient loading and sustained-release of HGF. HDC provides adhesion property as well as certain antioxidant ability, which works synergistically with HGF. The authors demonstrated the antioxidant and anti-inflammatory properties of GM@HDC@HGF in detail and proved its therapeutic effect in a rat Achilles tendinopathy model. Overall, this study is novel and interesting in this field and is recommended to be accepted after the following revisions.

1. In this study, HGF was used as a main therapeutic agent for tendinopathy. However, there are few detailed comments on HGF in the Introduction or Discussion sections. Relevant contents need to be added to compare and elaborate the role and advantages of HGF in the treatment of tendinopathy.

Response: Thank you very much for your comment. HGF is a multifunctional growth factor initially found in liver tissue that can effectively promote cell proliferation and regeneration. HGF is selected as therapeutic agents for tendinopathy in our study because it has shown efficacy in stimulating cell proliferation and matrix repair in chronic degenerative diseases such as osteoarthritis and intervertebral disc degeneration. Additionally, HGF have been found to mitigate inflammation by reducing the expression of COX-1 and COX-2 genes in tendinopathy. Furthermore, HGF can impede the differentiation of myofibroblasts, thereby reducing fibrosis and tissue adhesion. We have added a few explanations in the context. We have included some relevant content in the “Introduction” section. Please see below:

In this study, injectable methacrylate gelatin (GelMA) microspheres (GM) functionalized with dopamine and hepatocyte growth factor (HGF) were fabricated to inhibit oxidative stress and inflammation at the pathogenic sites of tendinopathy, regulate the local tendon microenvironment, ameliorate ECM metabolic disorders, and prevent tendon degeneration. The catechol groups in dopamine have excellent adhesion and antioxidant properties and can bind firmly to the surface of GM as well as to the affected tendons after in situ injection, making it available to perform its physiological functions [31]. HGF is a multifunctional growth factor initially found in liver with anti-inflammatory and matrix-regulation effects [32]. Studies have shown that HGF can promote proliferation and migration of tendon-derived stem cells and mitigate inflammation in injured tendons [33, 34]. Additionally, as a heparin-binding growth factor, HGF can form stable bonds with the heparin domain to avoid inactivation by external interference [35, 36]. Taking advantage of this, dopamine was combined with heparin via an amidation reaction to form the heparin-dopamine conjugate (HDC), which was used as a bridge to efficiently bind HGF to GM. In addition, the porous properties of GM ensured a high drug loading rate.

2. The authors need to show the specific sources of some materials and reagents for the convenience of readers. For example, the catalog numbers of antibodies.

Response: Thank you for your comments. We have included the corresponding item numbers of several important reagents in the “Materials and reagents” section in the revised manuscript. Please see blow:

All chemical reagents, unless mentioned otherwise, were purchased from Aladdin. Phosphate-buffered saline (PBS), DMEM/F12, fetal bovine serum (FBS), penicillin and streptomycin were purchased from Gibco. The recombinant rat HGF protein (80429-RNAH) was purchased form Sino Biological. A cell live/dead staining kit, a cell counting kit-8 (CCK-8) and a 2,7-Dichlorofluorescein diacetate (DCFH-DA) probe were purchased from Yeason. Anti-Nrf2 antibody (16396-1-AP), anti-HO-1 antibody (10701-1-AP), and anti-NQO1 antibody (67240-1-Ig) were sourced from Proteintech. Anti-COL-1 antibody (ab260043), anti-COL-3 antibody (ab7778), anti-MMP-3 antibody (ab53015), anti-MMP-13 antibody (ab39012), anti-iNOS antibody (ab178945), anti-IL-1β antibody (ab205924), anti-TNF-α antibody (ab9739), and anti-IL-6 antibody (ab233706) were sourced from Abcam. Primers for quantitative real-time polymerase chain reaction (qRT-PCR) were sourced from Generay. Enzyme-linked immunosorbent assay (ELISA) kits were sourced from Solarbio.

3. The authors isolated primary tenocytes from rat Achilles tendon tissues for in vitro experiments. However, tendon tissue contains other cells such as TSPCs. The absence of identification of tenocytes should be noted as a limitation.

Response: Thank you for this helpful suggestion. We referred to several relevant literatures for the isolation of tenocytes. Although the possibility does exist that the isolated cells contain other types of tendon cells, such method is the most commonly used protocol. Considering that tenocytes are the most abundant cells in tendon tissues, the isolated primary cells are generally recognized as tenocytes. It is regretful that we did not further characterize and classify the isolated primary cells. We will consider it in future studies.

4. In Figure 5, the difference between COL-3 immunofluorescence images does not seem to be significant. Please provide the corresponding intensity analysis of COL-1 and COL-3 images in a supplementary figure.

Response: Thank you for your suggestion. We have included the statistical analysis of collagen fluorescence intensity in Supplementary figure S5b. Please see below:

**Supplementary Figure S5.** a) Semi-quantitative analysis of expression of pro-inflammatory cytokines, ECM degrading enzymes, and ECM proteins. b) fluorescence intensity of COL-1 and COL-3. (n = 3, */**/***, #/##/### and $/$$/$$$ indicated *p* < 0.05/*p* < 0.01/*p* < 0.001 compared to the Control, LPS and GM@HDC@HGF groups, respectively).

5. There is obvious difference between the background colors of HE staining images in Figure 6 and Figure 7. Why did this happen? Please comment on that.

Response: Thank you for your careful review on our study. In fact, the tendon samples were stained and photographed at different times due to the different intervention time of two in vivo experiments. Subtle differences in staining steps and photography may have led to differences in background colors. We have adjusted the color difference of the images. Sorry again for the mistake.

6. For what reason did the authors choose the Bonar score to evaluate the severity of tendon injuries? What are the criteria for the Bonar score?

Response: Thank you very much for the questions. The Bonar scoring system is commonly used to classify the histopathological finding of tendinopathy. The variables included in the Bonar scale are (1) tenocytes; (2) ground substance; (3) collagen; and (4) vascularity. A four-point scoring system is used, where 0 indicates a normal appearance and 3 a markedly abnormal appearance. Overall, the total score for a given

slide could vary between 0 (normal tendon) and 12 (most severe abnormality detectable).

7. Why the authors chose 1-week and 4-week post-intervention time points for further in vivo experiments? Please comment.

Response: Thank you for your question. It was reported that in collagenase-induced rat Achille tendinopathy model, the acute phase occurred form day 3 to day 15, and then progressed towards the proliferative phase from day 30 and day 45 displaying a degenerative appearance associated with a very mild remodeling process. Thus, we chose the two time point to respectively study on the roles of modified microspheres in the acute and degenerative phases of tendinopathy.

8. There are some writing errors, including letter case and grammar errors. Please improve the context in detail.

Response: Thank you very much for your comment. We have submitted our manuscript for proofreading by a native English-speaker.

**Reviewer #5:**

Tendinopathy is a common disorder that causes local dysfunction and reduces quality of life. Recent research has indicated that alterations in the inflammatory microenvironment play a vital role in the pathogenesis of tendinopathy. Herein, injectable methacrylate gelatin (GelMA) microspheres (GM) were fabricated by the authors and loaded with heparin-dopamine conjugate (HDC) and hepatocyte growth factors (HGF). GM@HDC@HGF were designed to balance the inflammatory microenvironment by inhibiting oxidative stress and inflammation, thereby regulating extracellular matrix (ECM) metabolism and halting tendon degeneration. Combining growth factors with heparin was expected to improve the encapsulation rate and maintain the long-term efficacy of HGF. In addition, the catechol groups on dopamine have adhesion and antioxidant properties, allowing potential attachment at the injured site, and better function synergized with HGF. GM@HDC@HGF injected in situ in rat Achilles tendinopathy (AT) models significantly down-regulated oxidative stress and inflammation, and ameliorated ECM degeneration. In conclusion, the multifunctional platform developed presents a promising alternative for the treatment of tendinopathy. Overall, this manuscript is well-written and presents new and interesting information. There are some concerns as follows:

1) There is very limited comparison of the current results to existing literature. Microspheres based drug delivery has been attempted before and the current results should be compared to existing strategies.

Response: Thank you very much for your comments. It is regretful that we did not well compare our results with other relevant papers. In our study, we mainly compared GelMA microspheres with or without HDC modification. As we all know, GelMA can effectively load various growth factors. However, simple combination may result in the rapid release of growth factors. We chose HDC as a bridge between GelMA microspheres and HGF. On the one hand, HDC can be firmly loaded with HGF by electrostatic action and adhere to the surface of microspheres. On the other hand, the catechol groups on HDC exhibit antioxidant properties. Our results demonstrated that based on microsphere carriers, HGF can act synergistically with HDC in the treatment of tendinopathy.

2) Why did the authors use tenocytes instead of other tendon-derived cells for biocompatibility testing? Why not conducting the corresponding in vivo experiments? Response: Thank you for your comment. Tenocytes are the most numerous and representative cells in tendon tissues. Actually, the primary cells isolated from tendon tissues may also contain a small part of other tendon-derived cells, but we did not perform further cellular identification. In addition, due to the lack of experimental equipment, we were not able to conduct the corresponding experiments in vivo, such as microspheres degradation test.

3) Was tendon-like formation in regions where tendon formation would be undesirably observed? For example, was the surrounding connective tissues examined in each case for potential regenerative behavior given the inclusion of HGF in the microspheres? If this was not examined, then mention of the fact that off-target effects were not examined should be acknowledged as a limitation.

Response: Thank you very much for the question. In fact, Tendon regeneration relies primarily on the tendon tissue itself, whereas HGF plays a regulatory and supportive role. HGF will not directly lead to tendonogenesis. During the course of tendinopathy, the peritendinous connective tissues also develops an inflammatory phenotype. One limitation of our study is that the injected microspheres may not have been able to have a homogenous effect on the injured site. We feel so sorry for the shortcomings.

4) The present study administered the microspheres by injection. How to ensure that all the microspheres were injected to the expected site?

Response: Thank you for your comment. In the in vivo experiments, we selected rats with similar age and size to minimize experimental errors. Drugs and microspheres were uniformly injected at the position of 5 mm above the heel. Considering that skin incision may lead to infection and increased inflammation, we did not perform post-incision injection which is more precise than percutaneous injection.

5) Why did the authors use Col-1 and Col-3 for representative markers of tendon injury? Detailed explanations should be presented.

Response: Thank you very much for your comment. ECM degeneration is the critical feature of tendinopathy. In the early stage, COL-3 is produced and acts as a "patch" to protect the injured tendons. During the tendon healing process, aligned COL-1 fibers gradually replace COL-3. The normal tendon healing process can be inhibited by inflammation or repetitive mechanical loading, with insufficient COL-1 to replace the excessive COL-3 produced, ultimately exacerbating tendinopathy. Thus, the ratio of the two collagens is often used to evaluate the prognosis of tendinopathy.

6) Why did not the authors present the macro graphs of Achilles tendons after 1-week treatment for comparison?

Response: Thank you for very much for the observation. We feel sorry for the shortcoming. At the very beginning, we did not realize that the photographs of the gross appearance of rat Achilles tendon tissues should be retained. Because of that, we only presented the macro graphs of tendons after 4-week treatment. Sorry again for the omission.

7) The authors used two in vitro models for anti-inflammatory and antioxidant experiments. Is that necessary? LPS can also cause ROS change as reported in some studies.

Response: Thank you very much for your comment. Indeed, LPS has also been used as an intervention in oxidative stress models in various studies. However, according to our tests, changes in the corresponding indicators of oxidative stress were not evident in LPS-induced tenocytes. We hypothesize that this is due to the poor stimulus sensitivity of the primary cells. Therefore, we chose to use hydrogen peroxide to induce oxidative stress damages in tenocytes.
